# Peer-led family-centred problem management plus for immigrants (PMP-I) for mental health promotion among immigrants in USA: protocol for a pilot, randomised controlled feasibility trial

**DOI:** 10.1136/bmjopen-2022-061353

**Published:** 2022-04-28

**Authors:** Kalpana Poudel-Tandukar, Cynthia S Jacelon, Christopher R Martell, Krishna C Poudel, Shan Rai, Razu Ramdam, Holly Laws, Jerrold S Meyer, Elizabeth R Bertone-Johnson, Steven D Hollon

**Affiliations:** 1 Elaine Marieb College of Nursing, University of Massachusetts Amherst, Amherst, Massachusetts, USA; 2 Department of Psychological and Brain Sciences, College of Natural Sciences, University of Massachusetts Amherst, Amherst, Massachusetts, USA; 3 Department of Health Promotion and Policy, School of Public Health and Health Sciences, University of Massachusetts Amherst, Amherst, Massachusetts, USA; 4 Bhutanese Christian Society of Western Massachusetts, Westfield, Massachusetts, USA; 5 Department of Biostatistics and Epidemiology, School of Public Health and Health Sciences, University of Massachusetts Amherst, Amherst, Massachusetts, USA; 6 Department of Psychology, Vanderbilt University, Nashville, Tennessee, USA

**Keywords:** EPIDEMIOLOGY, MENTAL HEALTH, PSYCHIATRY, PREVENTIVE MEDICINE

## Abstract

**Introduction:**

Research is needed to investigate preventive strategies to reduce mental health burden and assess effective implementation among immigrants. Problem management plus (PMP) is a low-intensity multicomponent psychological intervention developed by the World Health Organization (WHO) that trained laypeople can deliver. PMP has been adapted as a prevention intervention and developed as PMP for immigrants (PMP-I), including psychoeducation, problem-solving, behavioural activations and mind–body exercise, to address immigrants’ multiple stressors. This pilot trial aims to assess the feasibility and acceptability of PMP-I and provide a preliminary estimate of the difference between PMP-I versus community support services pamphlets on the primary outcomes of interest (stress, anxiety and depressive symptoms) to inform the design of a large-scale intervention.

**Methods and analysis:**

The feasibility and acceptability of PMP-I will be assessed by measuring recruitment, session attendance, retention rates, programme acceptability and the fidelity of intervention delivery. This pilot trial will test preliminary effects of PMP-I vs community support services pamphlets in a randomised controlled trial (N=232 participants from 116 families (2 members/family); 58 families randomised to condition intervention or control) on stress, anxiety and depressive symptoms (primary outcomes), chronic physiological stress assessed in hair cortisol (secondary outcomes), and coping, family conflict resolution, and social networking (targets), with assessment at baseline, postintervention and 3-month postintervention. Eligibility criteria for the primary study participants include Bhutanese ≥18 years resettled in Massachusetts with a score of ≤14 on the Patient Health Questionnaire-9. All family members will be invited to participate in the family-based intervention (one session/week for 5 weeks). Multilevel modelling will compare the longitudinal change in outcomes for each treatment arm.

**Ethics and dissemination:**

The Institutional Review Board of the University of Massachusetts Amherst approved this study (Protocol: 1837). Written informed consent will be obtained from all participants. The study results will be used to inform the design of a large-scale intervention and will be disseminated in peer-reviewed journals and conferences.

**Trial registration number:**

NCT04453709.

**Strengths and limitations of this study**

- Whereas existing mental health interventions for immigrants are primarily based on treatment models to improve the access and quality of care for those with diagnosed mental health problems, this study is focused on developing, implementing, and pilot testing the effect of a culturally tailored preventative behavioural intervention to reduce stress and prevent mental health problems among immigrants.
- This study includes culturally tailored psychoeducation, behavioural activation, problem-solving and mind–body interventions that could help to address multiple psychosociocultural stressors through revitalising resources at the individual, family and community levels.
- The proposed intervention will be delivered to participants in their family environment by interventionists from the same community they trust and understand their language and problems from their cultural lens.
- This study will be among the first to link a preventive intervention with both biomarkers of stress (hair cortisol) and perceived stress and, using longitudinal data, to examine change over time in stress.
- Though clinical diagnosis is the gold standard, such an approach is not feasible in community-based studies, so this study relies on self-report measures of anxiety and depressive symptoms.

## Introduction

Refugees resettled in USA are vulnerable to mental health problems,^1 2^ such as anxiety and depression due to stress resulting from integrating into a new culture.^3–5^ Refugees’ risk for mental health problems increases during their acculturative process due to exposure to multiple stressors, such as adjustment to a new culture with limited language and sociocultural skills, perceived discrimination, and a lack of culturally mediated and protective social support resources.^5 6^ Although mental health treatments are available to help alleviate the intrapersonal, social, and economic costs of mental disorders, refugees greatly underuse these services.^1 7 8^ Thus, evidence-based, culturally tailored preventative mental health interventions are needed for the growing number of refugees in USA.

Existing interventions are focused explicitly on treatment models to provide quality care for those with diagnosed mental health problems^9^ that do little to help reduce stress and prevent mental disorders for those who have not yet developed diagnosable symptoms. For prevention, a culturally tailored intervention that addresses multiple psycho-socio-cultural stressors, including social and cultural integration, holds good promise.^10 11^ Community-based preventative interventions that promote positive impacts of social and cultural behaviours on mental health outcomes by protective resources are needed for the growing number of refugees dealing with life complexities.^12 13^ A review of community-based mental health interventions in refugees resettled to USA suggests^14^ that counselling, health promotion and skill-building workshops facilitated by refugee peers^15–17^ are helpful to reduce the psychological distress of many refugees who may be struggling with individual or family difficulties. Specifically, the Centers for Disease Control and Prevention recommends using a non-clinical, community support approach to prevent mental illness among refugees resettled in USA.^18^

Problem management plus (PMP) is a low-intensity evidence-based psychological intervention developed by the WHO that trained laypeople can deliver.^19 20^ PMP systematically teaches four strategies: stress management through mind–body exercises,^21–24^ problem-solving,^25^ behavioural activations^26–33^ and skills to strengthen social support for individuals with psychological distress. PMP has been proven successful in reducing depression for women with mental disorders in Pakistan in a group setting.^34^ We have adapted PMP to develop our PMP for immigrants (PMP-I) following a successful result of a pilot social and emotional well-being intervention. The pilot intervention included psychoeducation, mind–body exercise, problem-solving and social support and reduced more than 50% prevalence of anxiety and depression from preintervention to postintervention among Bhutanese refugees when delivered in either a group^35^ or a family setting.^36^ While promising, these pilot results were drawn from only those receiving the treatment; no control group was available for comparison. Thus, this study is to apply the adapted PMP in a randomised controlled trial (RCT). This study is indicated for several reasons: our intervention model demands integration of social and emotional stressors; promising results of PMP in a non-controlled pilot study; the need to test the efficacy of PMP using the more rigorous RCT study design; strong evidence of family and community ties in healthcare process; and growing consensus among community, scientists and policy-makers on the need for family-based care models that are sustainable.

## Objectives and hypothesis

The main objectives of this study are:

1. To assess the feasibility and acceptability of PMP-I: (1) recruitment, session attendance, retention rates, and programme acceptability; (2) feasibility of measures for assessing inclusion/exclusion and fidelity of intervention delivery and (3) barriers and facilitators of intervention using interview and focus group discussion (FGD) with participants and facilitators.
2. To test the preliminary outcomes of PMP-I among Bhutanese adults 18 years or older living in Massachusetts with a score of 14 or below on the Patient Health Questionnaire-9 (PHQ-9) with trained community facilitators. Our central hypothesis is that PMP-I will reduce stress, anxiety and depressive symptoms. We will test preliminary effects of PMP-I versus community support services pamphlets in a randomised pilot trial (N=232 participants from 116 families (2 members per family); 58 families per intervention and control) on perceived stress,^37^ anxiety and depressive symptoms (primary outcomes).^38^ chronic physiological stress assessed in hair cortisol (secondary outcome) and self-efficacy,^39^ coping,^40^ family conflict resolution,^41^ family satisfaction,^42^ social support^43^ and social networks (targets)^44^ with assessments at baseline, postintervention and 3-month postintervention.

## Methods and analysis

### Design and setting

This study will be conducted among resettled Bhutanese adults living in Massachusetts. Since 2008, Bhutanese people have been resettled in various states of the USA and are one of the largest groups of South Asian refugees (about 90 000).^45^ They bear a high burden of mental health problems both in the nation (depression: 20%; suicide rate: 21.5 per 100 000)^18^ and in western Massachusetts (depression: 24.0%; anxiety: 34.2%).^46^ Given the importance of family relationships, communication, and coping in mental health,^47^ the preventative social and emotional well-being intervention was designed for resettled Bhutanese adults in western Massachusetts using a community-based participatory research approach.^35^

**Figure 1:**
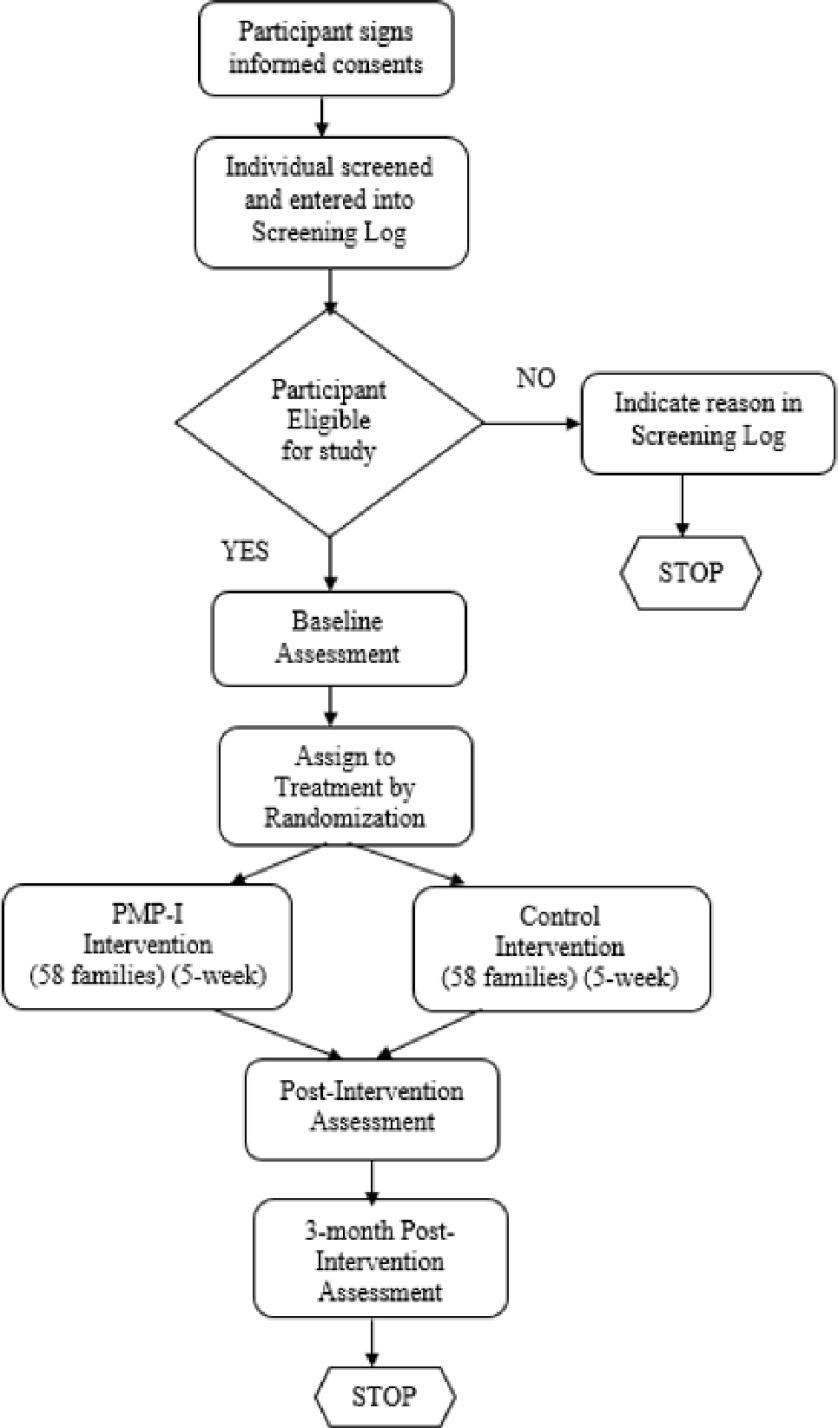
Study flow chart. PMP-I, problem management plus for immigrants.

This mixed-methods study will incorporate a two-arm randomised controlled feasibility trial and qualitative evaluation of PMP-I intervention’s acceptability to a range of stakeholders. The study protocol has been reported following the Standard Protocol Items: Recommendations for Interventional Trials. Figure 1 shows the study flowchart, and table 1 shows an overview of study measures.

**Table 1:** Overview of study measures

Assessment	Screening Visit 1	Baseline, Enrolment Randomisation: Visit 1	Intervention Session 1 Visit 2	Intervention Session 2 Visit 3	Intervention Session 3 Visit 4	Intervention Session 4 Visit 5	Intervention Session 5 Visit 6	Postintervention Visit 7	3 months Follow-up Final Visit
Informed consent form	X								
Screening tool	X								
Inclusion/exclusion criteria	X								
Demographics		X						X	X
Blood pressure		X						X	X
Bodyweight and height		X						X	X
Waist circumference		X						X	X
Hair samples		X							X
Stress, anxiety and depression		X						X	X
Family and social support		X						X	X
Coping strategies		X						X	X
Self-efficacy		X						X	X
Family conflict resolution		X						X	X
Family satisfaction		X							
Enrolment/randomisation		X							
Intervention session and its assessment using fidelity form			X	X	X	X	X		
Adverse events		X	X	X	X	X	X	X	X

### Participant recruitment

#### Participant inclusion criteria using a screening measure

This study will include eligible parents and adult children aged 18 and above interested in participating as primary study participants. At baseline, we will use a screening tool to identify individuals without significant depressive symptoms, as we aim to evaluate the effect of our intervention to prevent depression rather than treat depression. Eligibility criteria for our primary study participants include Bhutanese adults 18 years or older (both parents and children of each family) resettled in Massachusetts with a score of 14 or below on the PHQ-9, a screening questionnaire for depression. Our statistical analysis will focus on data from primary study participants only with baseline PHQ-9 scores 14 or below. However, all other interested adult family members, both parents and their adult children, regardless of PHQ-9 score, will be invited to participate in the intervention. The PHQ-9 scores of participants will not be disclosed to anyone to maintain individual confidentiality. Besides, individuals with PHQ-9 screening scores ‘15–19’ (moderately severe depression) and ‘20–27’ (severe depression) will be provided with feedback on their screening questionnaire outcomes confidentially. They will be encouraged to consult their primary healthcare providers.

#### Participant exclusion criteria

Participants with clinically diagnosed mental disorders and those taking psychiatric medications for any mental health problems will also be encouraged to participate in the family-based intervention activities. However, in our primary statistical analysis, we will not consider data from those participants with PHQ-9 scores of 15 or above or diagnosed with mental health problems.

### Informed consent

The principal investigator (PI) has prepared an informed consent document including an explanation on study background, screening, recruitment criteria, sample size, data collection and intervention, study risks and benefits, confidentiality, National Institute of Mental Health Data Archive (NDA) data sharing policy, and hair samples collection procedure (online supplemental file 1). Trained community research assistants (RAs) will inform screening and study procedures to each participant using UMass Amherst Institutional Review Board (IRB)-approved single informed consent form visiting in-person. Once participants understand study details, RAs will request their signature or initials or fingerprint for those who cannot write in the consent form before data collection. Participants will be reminded that their participation in the study is voluntary and free to leave the study without penalty.

10.1136/bmjopen-2022-061353.supp1 Supplementary data

### Sample size and power calculations

The goal of the pilot project is to estimate the magnitude of the difference between the preventive intervention and the education control on the primary outcomes of interest to inform the design of a large-scale intervention. We conducted a power analysis to detect an effect size (ES) as small as ES=0.30 with alpha=0.05 and power of 0.80. We may find a larger effect in our pilot, but our understanding is that power estimates should be based on the smallest effect we want to detect rather than the size of the effect that we expect.^48^ Analyses were performed using Optimal Design^49^ by accounting for the intra-correlation among family members of 0.10 and alpha=0.05, we would have 80% power to detect a standardised difference of ES=0.30 between two treatment groups of 116 families (58 per treatment arm) with an equal probability of being randomised to each of our two intervention arms.

### Randomisation

We will randomly allocate selected families into intervention and control groups using a random sampling method after the baseline survey. RAs are unaware of which group the family will be randomised to when collecting baseline data. We will randomly assign 116 interested families (58 families per intervention and control) using a random number table. For random allocation, first, the PI will prepare the sampling frame that lists interested families, then assign a number to each family in the sampling frame, and finally select 116 numbers using a table of random numbers. We will assign a random number selected at the first attempt for intervention and the second attempt for control.

Procedures are in place for tracking the participants for intervention and follow-up (eg, contact address and phone). RAs will visit selected families and brief them about study procedures, informed consent, and procedures to protect human subjects. Two adult members of the selected families who meet the inclusion criteria and give informed consent will be recruited for the study. We will follow up with all families randomised to either study arm. We will not follow up with participants if they decide to end their participation at a particular time point of our study. But, we will include their already collected data in our analysis. Given our strong community networks and mobilisation of community RAs, we anticipate low attrition rates in practice.

### PMP for immigrants

PMP-I is a 5 week, peer-led, culturally tailored mental health promotion programme that includes psychoeducation, problem-solving, behavioural activation (90 min), breathing exercises, and yoga (90 min) in a family setting. PMP-I will use a structured approach, including once-a-week face-to-face sessions, yoga practice, breathing exercises, homework that includes practice activities, rebuilding individual skills, or learning new skills to reduce stress.

Our intervention aims to develop skills in coping adaptively in a new culture, seeking help and support for mental health problems, and other life skills opportunities that can improve their quality of life. Module 1: Managing stress includes yoga, breathing exercise, stress-management sessions, and practice exercises to develop coping strategies that are most helpful to reduce stress and then plan a strategy to carry out those solutions. Module 2: Managing problems includes practice exercises to identify the problems causing the most concern, develop solutions that are most helpful in addressing the problem, and then plan a strategy to carry out those solutions. Module 3: Behavioural Activation includes communication skill sessions and practice exercises to identify pleasant activities (time to yourself; connecting with others; self-care), breaking down the task into smaller steps and schedule tasks, and then plan a strategy to carry out those tasks. Module 4: Strengthening social support includes social skills sessions and practice exercises to identify at least one person or service from whom the participant feels comfortable getting some support, and to plan exactly what the participant is going to do, and then schedule a day to carry out the tasks. Module 5: Staying well includes practice exercises to make a plan that helps to create a supportive family environment.

Community interventionists (CIs) are trained community members with at least a high school level of education, and no formal training or prior experience with mental health will deliver the PMP-I. Dr. Christopher Martell, board-certified in behavioural and cognitive psychology and clinical psychology and a Massachusetts Licensed Psychologist, will provide 12 days of training to the interventionists in collaboration with the PI and Dr. Steven D. Hollon (Professor of Psychiatry, Psychology and Human Development) following the WHO PMP Helpers’ Training Guide^50^ adapted for PMP-I. Classroom training includes information about stress, depression, mental health problems, the rationale for each intervention strategy, necessary helping skills, practice plan formulation, role-plays, peer observations, and group discussion related to core intervention concepts, practices and supervision skills. Supervision involves discussing participants’ progress and difficulties experienced when delivering strategies and role-playing on managing problems or practising skills. They will use the PMP-I intervention manual to provide PMP-I to community members in family settings under field supervisors and PI’s supervision. We will conduct a formal evaluation of the interventionists' readiness to implement/supervise the PMP-I intervention, such as using the manual, answering questions, managing time, using a fidelity checklist, practising exercise and role play to provide feedback as necessary.

A licensed yoga trainer will provide 4 hours of breathing exercises and 16 hours of yoga to CIs and field supervisors using a mind–body exercise training manual. Classroom training includes theoretical and practices to guide participants in mind–body exercises for attention to breath, body sensation, emotional awareness, and mental function on different postures of yoga practices such as Pranayama (3 poses) and Asana (21 poses). Training will include practice assessment at the end to ensure that all field staff is trained, using a checklist, practice exercise and role-plays.

### Community support service programme

Bhutanese community members expressed that knowing the health and life skill development programme available in their communities would benefit them in strengthening their life skills.^51^ By considering their request, we have prepared pamphlets including names, contact, and service details of community and health organisations in the area where they live. CIs will distribute community support service programme pamphlets to control families.

### Feasibility and acceptability assessment

The PMP trainer’s training guidelines provide specific tools for evaluating and monitoring the intervention, which we use to monitor intervention delivery fidelity. These tools are PMP Quiz, PMP Helper’s Supervision Form, PMP Helper Classroom-based Competency Assessment, PMP Helper In-field based Competency Assessment, PMP Trainer/Supervisor Competency Assessment and Session-by-Session Checklists for PMP Helpers.^50^ We have adapted these tools in the context of our programme contents. Using these standard tools, we will evaluate session-by-session classroom and in-field based competencies of CIs and field supervisors and provide them feedback as needed using supervision forms, role-plays, group discussion and training.

At the field level, field supervisors will monitor intervention sessions delivered by CIs using standard checklists. Items include adherence to the manual, per cent of intervention content administered, proper use of time/materials and adequate response to participants’ questions. They will also monitor participants’ engagement, acceptability and satisfaction via brief questionnaires with participants and interventionists during and after intervention completion. Moreover, CIs will be asked to complete a structured checklist on the attendance, compliance and satisfaction towards intervention components immediately after each session.

The PI will conduct an FGD in the Nepali language with interventionists, supervisors, and participants separately to collect information on barriers and facilitators of intervention, perceptions about whether the intervention met participants' needs, and feedback on how effectively the programme team worked with participants. Interviews and FGD will be documented verbatim in a written transcript for subsequent analysis. All qualitative data will be analysed using thematic content analysis.^52^ Feedback provided by the field staff will be reviewed and coded to identify recurrent themes regarding the intervention’s acceptability. Fidelity data will be used to assess intervention content and transmission.

### Primary outcome measures

#### Anxiety and depressive symptoms

The Hopkins Symptom Checklist-25 (HSCL-25) will be used to measure anxiety, and depressive symptoms experienced over the past month.^38^ It is composed of a 10-item subscale for anxiety and a 15-item subscale for depression, with each item scored on a Likert scale from 1 (not at all) to 4 (extremely). The scale has high internal consistency (Cronbach’s α) for anxiety (0.95) and depression (0.94) in the Bhutanese study.^35^

#### Perceived stress

The 10-item Cohen Perceived Stress Scale (PSS) will be used to assess perceived stress.^37^ The PSS uses a 5-point Likert scale (ranging from 0, ‘never’ to 4, ‘very often’) to assess psychological stress experienced during the past month, including the extent to which situations felt unpredictable, uncomfortable and overwhelming. In the Bhutanese study, the scale has high internal consistency (Cronbach’s α=0.80).^35^

### Secondary outcome measures

#### Physiological stress

We will use the ELISA cortisol hair test (average hormone levels over the past 3 months) as a biomarker to measure physiological stress. Hair samples will be processed in the neuroendocrine lab at the University of Massachusetts Amherst.^53 54^ Sensitive and specific enzyme immunoassay (Arbour Assays) will be used for the analysis. The assay has intraassay and interassay coefficients of variation of <10%.

### Other measures

#### Coping strategy

Coping strategy will be measured using a 32-item Coping Strategies Inventory-Short Form (CSI-SF).^40^ The CSI-SF includes two overall coping factors, Engagement and Disengagement, and four secondary factors, Problem Engagement, Problem Disengagement, Emotion Engagement, and Emotion Disengagement. The CSI-SF scale (Cronbach’s α=0.95) has high internal consistencies in the Bhutanese study.^35^ Participants were asked to rate their responses on a 5-point Likert-type scale ranging from not at all (1) to very much (5).

#### Coping self-efficacy

Self-efficacy will be measured using a 26-item Coping Self-efficacy scale for coping with challenges and threats.^39^ Each item of the scale will be rated on an 11-point scale Likert-type scale ranging from (0) cannot do at all, (5) moderately certain can do, and (10) certain can do. The scale has high internal consistency (Cronbach’s α=0.96) in the previous Bhutanese study.^35^

#### Social support

Perceived social support will be measured using a 12-item Multidimensional Scale of Perceived Social Support,^43^ including support from friends, family, and significant others. A sample item for this scale is, ‘My family tries to help me’. Each item of the scale will be rated on a 5-point Likert-type scale ranging from strongly disagree (1) to strongly agree (5). Graded items will be summed up to provide a total score, and higher scores indicate high social support. The scale has high internal consistency (Cronbach’s α=0.92) in the previous Bhutanese study.^35^

#### Social network

We will use a Lubben Social Network Scale-Revised to measure social networks among family and friendships.^44^ It consists of six questions, which assess kinship ties, and a comparable set of six questions, which determine friend ties by replacing the word relatives with the word friends. We prepared three questions to measure cross-cultural social ties following a similar pattern. The scale has high internal consistency for kinship ties (Cronbach’s α=0.78), friendship ties (Cronbach’s α=0.80), and cross-cultural social ties (Cronbach’s α=0.74) in the previous Bhutanese study.^35^ These items will be scored on a five-point Likert scale ranging from none (0) to 9 or more or always (5).

### Family conflict resolution

Family conflict resolution, including positive or negative resolution, effective communication and discussion of differences, will be measured using a 17-item version of the ‘Family Conflict Resolution’ scale.^41^ This scale has high internal consistency (Cronbach’s α=0.90) in the previous Bhutanese study.^35^ Participants will be asked to respond on a 7-point Likert-type scale, ranging from never (1) to always (7).

#### Family satisfaction

Family satisfaction with various aspects of family functioning, including family closeness, flexibility and communication, will be measured using a 10-item family satisfaction scale.^55^ Participants will be asked to respond on a 5-point Likert-type scale, ranging from very dissatisfied (1) to extremely satisfied (5).

### Data management

All interviews will be conducted with the utmost privacy and confidentiality. Each interested and eligible adult participant in the family will be interviewed individually, in a private place where they feel comfortable, by our trained community RAs. The RAs will ensure audio and visual privacy at these sites, and ensure data confidentiality. RAs will reassure participants that numerical codes would be used in place of names in all records to ensure confidentiality. The survey materials (questionnaires, transcriptions, and field notes) will be stored in a locked cabinet in the PI’s office. Data entry will be done on the PI’s office computer (encrypted and password protected) under the full supervision of the PI. The original data will be kept on OneDrive, a secure, networked university data storage system. De-identified data sets will be used for statistical analyses. The PI herself will do data analysis and documentation. All information will be presented in aggregate form in the manuscript or conference abstract, and no individual respondent will be identified.

### Data analysis

We will compare baseline characteristics of intervention and control groups using χ^2^ and t-tests as appropriate. While differences between groups are not expected because of the randomisation used in the study design, variables showing significant differences between the two groups will be included as covariates in primary analyses. The primary analyses will test whether participants’ outcomes in the PMP-I arm differ from those in the control arm. Multilevel modelling will compare outcomes of each treatment arm while accounting for the clustering of participants within families. Continuous outcomes will be analysed using hierarchical linear modelling, and dichotomous outcomes will be analysed using multilevel generalised linear models with a Bernoulli distribution appropriate to nonlinear binary outcomes.^56^

We expect approximately 2–4 members for each of the 58 families in each treatment arm, and the correlation among family members’ responses will be accounted for in the model. Hierarchical or multilevel modelling is suited to these data as it accounts for the clustering of members within families and unbalanced designs (ie, different family sizes).^56^ This will be an intention-to-treat type of analysis, as multilevel modelling allows retention of all participants irrespective of the number of sessions attended (multilevel modelling uses maximum likelihood estimation, one of the recommended ways of handling missing data). The analysis will estimate endpoint outcomes based on repeated measures (level 1) within individuals (level 2) within families (level 3). Separate models will be created to evaluate the relationships between mediators (targets) and outcomes and explore mediators (eg, coping) of intervention-outcome relation. All analyses will be performed using SAS, V.9 (SAS Institute).

### Independent safety monitor

We will select an independent safety monitor (ISM) with mental health expertise, whose primary responsibility is to provide independent monitoring of this clinical trial in a timely fashion. Overall, the ISM will review enrollment data, safety data, and data integrity to maintain safety in the trial. The PI will submit data reports once a year to the ISM. The report will include the key variables necessary for monitoring the safety and quality of data collection and the integrity of the study, including inclusion criteria, informed consent, subject enrollment and retention, data confidentiality, intervention compliance, dropouts, adverse events, protocol compliance, data quality and baseline characteristics of study participants. The ISM will have access to all safety and data quality information collected and will have the authority to stop the study if it is determined that there are unacceptable risks to participants. The ISM also will review the study protocol, informed consent, and all relevant documents before the onset of the study and will review and approve amendments to these documents. The ISM will issue a monitoring report to the PI following each review. The PI will submit all review reports to the UMass Amherst IRB and National Institute of Mental Health (NIMH) Programme Officer in annual progress reports.

### Trial management

The PI will assume overall responsibility of trial management, working together with the entire research team throughout the project, meeting monthly with Co-Investigators (psychiatrist, cognitive behaviour therapist and epidemiologist), and once every week with field staff (supervisors, interventionists and RAs) via in-person or zoom or text message as needed. During the trial, experienced field supervisors from the Bhutanese community, who are trained as a community health workers and have worked with the PI in previous family-based mental health intervention studies with depressive and suicidal ideation outcomes, will take responsibility for the day-to-day oversight of the participants and field teams in the implementation of the trial. Field supervisors will immediately report any noted adverse events among participants to the PI. The PI will report adverse events data to the ISM, UMass Amherst IRB, and NIMH Programme Officer following NIMH guidelines for reportable events, as described below.

### Adverse events reporting

Throughout the study period, all study participants will be monitored daily by the field supervisors under the PI’s supervision. Field supervisors will request study participants and their family members to immediately report any unanticipated serious adverse events in their family, such as (1) reporting suicidal ideation or attempts, hospitalisation, disability and/or death; (2) discomfort with the PMP-I programme content and/or evaluation procedures, and (3) risk of a breach of confidentiality, of the collected data and/or by programme personnel to field supervisor or PI directly. Field supervisors will immediately report details of such adverse events to PI. The PI will be responsible for reporting them to the UMass Amherst IRB, ISM and NIMH Programme Officer by secure email within ten business days of the study team becoming aware of any serious adverse events. The PI will be responsible for summarising all adverse events that are deemed expected and/or unrelated to the study in the annual progress report submitted to the UMass Amherst IRB, ISM and NIMH Programme Officer by secure email.

### Patient and public involvement

Patients or the public were not involved in the design, or conduct, or reporting, or dissemination plans of our research.

## Ethics and dissemination

The Institutional Review Board of the University of Massachusetts Amherst approved this study (Protocol ID: 1837) and certified that it will be performed according to the ethical standards laid down in the 1964 Declaration of Helsinki and its later amendments.

Before enrolling participants in the study, written informed consent will be taken from each person after a complete description of the study. All participants will have the opportunity to discuss any questions or issues (online supplemental file 1).

The study data will be shared via the NDA. Access to data used in the proposed project will be considered for sharing in compliance with the National Institutes of Health Grants Policy on Sharing Unique Research Resources. The study results will be used to inform the design of a large-scale intervention and will be disseminated in peer-reviewed journals and conferences.

## Discussion

This study reports an RCT protocol that tests PMP-I’s feasibility, acceptability and preliminary outcomes with trained community facilitators. This study is built on prior research that has shown the effectiveness of social and emotional well-being intervention, including psychoeducation, problem-solving, social support and mind–body exercises, to reduce stress, anxiety and depressive symptoms among Bhutanese adults resettled in MA at a group^35^ and family settings^36^ using a pre-test and post-test intervention design. Our project designed for Bhutanese immigrants includes evidence-based interventions of specific relevance to this community, such as psychoeducation,^57^ problem-solving,^25^ behavioural activations,^26–33^ mind–body exercises,^21–24^ and strengthening social support to address identified social (eg, social isolation, language difficulties) and emotional (eg, lack of self-esteem or self-efficacy)^51^ stressors by strengthening protective factors (eg, resilience or coping).^46 58^ This study is innovative as it will be the first culturally tailored, preventive, family-based, multicomponent behavioural intervention driven by the community to reduce stress. We will have pilot-tested a preventative mental health intervention for Bhutanese adults on completion. This study can be expected to impact reducing stress and promoting immigrants’ mental well-being.

Our project is likely to be replicated with other immigrant communities with minimal adaptation over the long term for three reasons. First, our intervention is guided by a strengths-based approach in which we plan to include community strengths. This principle can be applied to capture and integrate the strengths of any community. Second, our programme prioritises the training of community members as interventionists, as these are individuals whom the community trusts, who share the same cultural lens as the community and thus can well understand language and specific challenges, and who have a vested interest in the strength and resilience of their community. This aspect of our intervention design is easily adaptable to other populations. Finally, intervention is designed to be delivered in family settings where participants are most comfortable and family members can support each other throughout their lives. This component is crucial in collectivistic societies where family bonds and group identity are strong. Thus, our family-based strategies could be replicable in other immigrant groups, where there are similarities in social and emotional stressors, challenges, community strengths (coping, resilience, social support), strong family support, and cultural preference of native community counsellors for their mental health consultation. Our strength-based and peer-led strategies promote community engagement and make the intervention sustainable.^59–61^

Although our study design has several strengths, it also has some methodological limitations. We measured anxiety and depressive symptoms using the HSCL-25 scale, which was validated with clinical Diagnostic and Statistical Manual of Mental Disorder-4 (DSM-IV) diagnoses of major depressive disorder^62^ among refugees in Nepal^63^ and other countries.^64^ Although clinical diagnosis is the gold standard; such an approach is not feasible in community-based studies. We have started implementing an intervention during the COVID-19 pandemic, so we may need to be flexible in the time frame for conducting surveys and implementing intervention sessions while waiting for participants to recover from COVID-19 infections they might have contracted during the study period.

In conclusion, our trial will provide information on the feasibility of PMP-I among the immigrant population and ES estimate to design a larger-scale RCT intervention study.

### Trial status

Recruitment of participants was delayed due to the COVID-19 pandemic and started on 17 August 2021. At the time of manuscript submission, trial was ongoing. Results of this study are expected in mid-2024.

## Supplementary Material

Reviewer comments

Author's manuscript
