# Supplementary material for: Peer-led family-centred problem management plus for immigrants (PMP-I) for mental health promotion among immigrants in USA: protocol for a pilot, randomised controlled feasibility trial
Source: BMJ Open. 2022 Apr 28;12(5):e061353. doi: 10.1136/bmjopen-2022-061353 (PMC9097550; doi:10.1136/bmjopen-2022-061353)
Supplement: Supplementary data [file bmjopen-2022-061353supp001.pdf]

Supplemental material
BMJ Publishing Group Limited (BMJ) disclaims all liability and responsibility arising from any reliance placed on this supplemental material which has been supplied by the author(s)

Consent Form for Participation in a Research Study
University of Massachusetts Amherst

Researcher(s): Kalpana Poudel-Tandukar, Assistant Professor, College of Nursing
Study Title: Reducing stress, anxiety, and depressive symptoms via a family-centered preventative intervention for immigrants: A randomized controlled feasibility trial
Study Funder: National Institute of Mental Health, National Institutes of Health

---

1. WHAT IS THIS FORM?

"This form is called a Consent Form. It will give you information about the study so you can make an informed decision about participation in this research. We encourage you to take some time to think this over and ask questions now and at any other time. If you decide to participate, you will be asked to sign this form and you will be given a copy for your records."

2. WHAT ARE SOME OF THE IMPORTANT ASPECTS OF THIS RESEARCH STUDY THAT I SHOULD BE AWARE OF?

This study plans to implement mental health promotion program among Bhutanese adults resettled in Massachusetts through trained community interventionists in collaboration with church leaders. The overall objective of this study is to assess preliminary effect of a family-centered mental health promotion program (Problem Management Plus for Immigrants: PMP-I) to improve coping, family wellbeing, and social networking that would be helpful to reduce stress, anxiety, and depressive symptoms among Bhutanese adults resettled in Massachusetts. We will determine your eligibility by asking questions of Patient Health Questionnaire-9 (PHQ-9), a screening tool for depression. You are eligible to participate in this study as a primary study participant if your PHQ-9 score is 14 or below. If you are eligible and interested, we will take an informed consent prior to take about an hour interview before, after, and 3-month after program. We will request you to attend about 2-3 hours mental health promotion program each week for 5-week. You will be randomly assign into one of our program, PMP-I program or talk program using a community support service pamphlet. If you are assigned to talk program now, we will provide you PMP-I program after completion of 3-month follow up survey. You do not need to answer the questions, which you do not feel comfortable. Your information will be kept confidential and will not be documented with your name. Please find risks and benefits of study and other important details in the rest of the consent document.

3. WHY ARE WE DOING THIS RESEARCH STUDY?

Existing mental health interventions for immigrants are largely based on treatment models to improve the access and quality of care for those with diagnosed mental health problems, however culturally-tailored preventative behavioral interventions aimed at reducing stress and preventing mental health problems among immigrants are limited. Thus, we aim to develop, implement, and pilot test the preventive behavior intervention driven by community members to reduce stress and prevent mental health problems among immigrants.

4. WHO CAN PARTICIPATE IN THIS RESEARCH STUDY?

Bhutanese adult aged 18 years or older living in the Massachusetts with a score 14 or below on the Patient Health Questionnaire (PHQ-9) and are willing to participate in the study voluntarily will be requested to participate in the study as primary study participants. This includes parents and their adult children aged 18 and above. Bhutanese adults with PHQ-9 scores 15 or above or with clinically diagnosed mental disorders or taking psychiatric medications for any mental health problems will not be eligible to participate in this study as a primary study participant. However, all interested adult family members both

IRB OFFICE USE ONLY

University of Massachusetts Amherst-IRB
Protocol #: 1837
IRB Signature: Nancy C. Swett

parents and their adult children, regardless of PHQ-9 score and mental health status, will be encouraged to participate in our mental health promotion intervention at family settings.

5. WHERE WILL THIS RESEARCH STUDY TAKE PLACE AND HOW MANY PEOPLE WILL PARTICIPATE?

This study will be conducted among Bhutanese adults resettled in the Massachusetts at their family settings. We plan to enroll 232 participants from 116 families in this study. The entire study is expected to complete in three year time period.

6. WHAT WILL I BE ASKED TO DO AND HOW MUCH TIME WILL IT TAKE?

If you agree to take part in this study, we will take your 5-minutes time asking short questions of Patient Health Questionnaire, which is a screening tool for depression. If you are eligible to participate in this study, we will take your informed consent first. Then, we will take about 45 to 60 minutes of your time for questionnaire survey before and after program and 3-month after program (thrice). The questionnaire consists of questions related to socio-demographic information, depression, perceived stress, family satisfaction, coping behavior, family/social support, family wellbeing, social network, sleep quality, and health information and program assessment information. We will also measure your body weight, height, waist circumference, and blood pressure. We will also take your 3-cm hair sample before and 3-month after program (twice). We request you not to write your name in the questionnaire. You may skip any question you feel uncomfortable answering. We will also take about two to three hours of your time to attend mental health promotion program each week for 5-week.

7. WILL BEING IN THIS RESEARCH STUDY HELP ME IN ANY WAY?

You may not directly benefit from this research; however, we hope that your participation in the study may find useful learning about mental health promotion activities.

8. WHAT ARE MY RISKS OF BEING IN THIS RESEARCH STUDY?

We believe there are minimal risks associated with this research study; however, a risk of breach of confidentiality always exists and we have taken the steps to minimize this risk as outlined in section 9 below. Some of the questions that we ask may put you in trouble or you may hesitate to answer, for example, questions on suicidal intention. You are free to skip such questions or also withdraw yourself from participating the whole study. As researchers, we are not qualified to provide counselling services and we will not be following up with you after this study. If you feel upset during the study, or find that some questions or aspects of the study triggered distress, you may contact the PI. The PI will assist you (in your native Nepali language) and help you to connect with mental health support services in coordination with the field supervisor. You may want to contact your counsellor at nearby health institutions such as Baystate Medical Center (Phone: 413-794-0000) and Caring Health Center (Phone: 413- 739-1100), Springfield. Field supervisors from community will provide necessary support to you for setting up your appointment with primary health care providers if needed.

9. HOW WILL MY PERSONAL INFORMATION BE PROTECTED?

Your privacy and confidentiality is important to us. The following procedures will be used to protect the confidentiality of your study records. All the information collected during the study will remain confidential. We will conduct survey in a private location, only allowing authorized research team members to meet with research participants. We will not record your name in the questionnaire. We will assign you numerical code that would be used in place of your name in all records to ensure confidentiality. We would like to assure you that your information will be kept confidential and will not be disclosed in your name in any of our record, report, publications, and presentations. Data will be stored securely in password protected computer and will be made available only to the PI. The PI will keep all study records, including any codes to your data, in a password protected computer. All signed consent documents will be

IRB OFFICE USE ONLY

University of Massachusetts Amherst-IRB
Protocol #: 1837
IRB Signature: Nancy C. Swett

stored securely and separately from the research data in the separate locked cabinet. All hardcopies of study materials will be stored securely in the locked cabinet of PI's office. Research records will be labeled with a code. A master key that links names and codes will be maintained in a separate and secure location. The master key and questionnaires will be completely destroyed six years after the close of the study. All electronic files containing identifiable information will be password protected. Any computer hosting such files will also have password protection to prevent access by unauthorized users. Only the PI will have access to the passwords. At the conclusion of this study, we plan to publish the study findings. Information will be presented in summary format and you will not be identified in any publications or presentations.

The hair sample will be stored in a clean dry white envelope with your ID number on top of the envelope. The hair samples will be stored in the College of Nursing's laboratory during the data collection process. At the end of survey, the collected hair samples will be sent to the laboratory in UMass Amherst. Hair samples will be processed in the laboratory for cortisol measurement. If sample remains after measurement procedure completion, it will be disposed according to the safety rules and regulation of the laboratory.

For this study you will be assigned a global unique identifier (GUID). This GUID is generated as a subject ID that allows researchers to share raw data such as number or percentage specific to a study participant without exposing personally identifiable information. The GUID is made up of random alpha-numeric characters and is NOT generated from personally identifiable information or protected health information. This identifier will be kept separate from your paper consent file and will be stored in a password protected electronic file. Descriptive/raw data will be submitted semi-annually. Access to raw data used in the proposed project will be considered for sharing in compliance with the NIH Grants Policy on Sharing Unique Research Resources. Any raw data to be released for sharing will not contain identifiers (such as name, address, birthdate and phone number) of the study participants.

During and after the study, we will send deidentified study data to the National Institute of Mental Health Data Archive (NDA). Experts at the NIH who know how to keep your data safe. The study researchers will make every attempt to protect your identity. The study data provided to NDA may help researchers around the world learn more about mental health and how to help others who have problems with mental health. NIMH will also report to Congress and on its website about the different studies using NDA data. You will not be contacted directly about the study data you contributed to NDA. You may decide now or later that you do not want your study data to be added to the NDA. You can still participate in this research study even if you decide that you do not want your data to be added to the NDA.

This research is covered by a Certificate of Confidentiality from the National Institutes of Health. The researchers with this Certificate may not disclose or use information, documents, or hair samples that may identify you in any federal, state, or local civil, criminal, administrative, legislative, or other actions.

A description of this study will be available on http://www.ClinicalTrials.gov. This Web site will not include information that can identify you. At most, the Website will include a summary of the results, when they are available. You can search this Web site at any time. The registration number for this study is NCT04453709.

10. WHAT IF THERE IS AN UNEXPECTED FINDING ON TESTS CONDUCTED ON MY HAIR SAMPLES?

The investigators for this research project are not licensed or trained diagnosticians or clinicians. The testing performed in this project is not intended to find abnormalities, and the data collected do not comprise a diagnostic or clinical study thus we would not be returning/sharing the results of tests that are conducted on your hair samples.

11. WILL MY INFORMATION (HAIR SAMPLES OR PRIVATE INFORMATION) BE USED FOR RESEARCH IN THE FUTURE?

Your information or hair samples will not be used or distributed for future research studies even if identifiers are removed.

IRB OFFICE USE ONLY

University of Massachusetts Amherst-IRB
Protocol #: 1837
IRB Signature: Nancy C. Swett

12. WILL I BE GIVEN ANY MONEY OR OTHER COMPENSATION FOR BEING IN THIS RESEARCH STUDY?

You will be interviewed about 45 to 60 minutes for three times (before and after program and 3-month after program). We will provide total $25 cash for each participant after completion of each survey.
After baseline survey: $25 per individual
After post-intervention survey: $25 per individual
After 3-month follow up survey: $25 per individual
After intervention: $50 per family ($10 per session)
Exercise mat: One per individual

13. WHO CAN I TALK TO IF I HAVE QUESTIONS?

Take as long as you like before you make a decision. We will be happy to answer any question you have about this study. If you have further questions about this project or if you have a research-related problem, you may contact the researcher, [Dr. Kalpana Poudel Tandukar; email: kalpana@umass.edu; Tel: 1-413-545-5095). If you have any questions concerning your rights as a research subject, you may contact the University of Massachusetts Amherst Human Research Protection Office (HRPO) at 1-413-545-3428 or humansubjects@ora.umass.edu.

14. WHAT HAPPENS IF I SAY YES, BUT I CHANGE MY MIND LATER?

You do not have to be in this study if you do not want to. If you agree to be in the study, but later change your mind, you may drop out at any time. There are no penalties or consequences of any kind if you decide that you do not want to participate. You will be notified of all significant new findings during the course of the study that may affect your willingness to continue.

15. WHAT IF I AM INJURED?

The University of Massachusetts does not have a program for compensating subjects for injury or complications related to human subjects research, but the study personnel will assist you in getting treatment.

16. SUBJECT STATEMENT OF VOLUNTARY CONSENT

When signing this form I am agreeing to voluntarily enter this study. I have had a chance to read this consent form, and it was explained to me in a language which I use. I have had the opportunity to ask questions and have received satisfactory answers. I have been informed that I can withdraw at any time. A copy of this signed Informed Consent Form has been given to me.

____________________	____________________	__________
Participant Signature:	Print Name:	Date:

By signing below I indicate that the participant has read and, to the best of my knowledge, understands the details contained in this document and has been given a copy.

____________________	____________________	__________
Signature of Person Obtaining Consent	Print Name:	Date:

IRB OFFICE USE ONLY

University of Massachusetts Amherst-IRB
Protocol #: 1837
IRB Signature: Nancy C. Swett
