# Supplementary material for: Peer-led family-centred problem management plus for immigrants (PMP-I) for mental health promotion among immigrants in USA: protocol for a pilot, randomised controlled feasibility trial
Source: BMJ Open. 2022 Apr 28;12(5):e061353. doi: 10.1136/bmjopen-2022-061353 (PMC9097550; doi:10.1136/bmjopen-2022-061353)
Supplement: Author's manuscript [file bmjopen-2022-061353.draft_revisions.pdf]

BMJ Open is committed to open peer review. As part of this commitment we make the peer review history of every article we publish publicly available.

When an article is published we post the peer reviewers' comments and the authors' responses online. We also post the versions of the paper that were used during peer review. These are the versions that the peer review comments apply to.

The versions of the paper that follow are the versions that were submitted during the peer review process. They are not the versions of record or the final published versions. They should not be cited or distributed as the published version of this manuscript.

BMJ Open is an open access journal and the full, final, typeset and author-corrected version of record of the manuscript is available on our site with no access controls, subscription charges or pay-per-view fees (http://bmjopen.bmj.com).

If you have any questions on BMJ Open's open peer review process please email info.bmjopen@bmj.com

BMJ Open

Peer-led family-centered Problem Management Plus for Immigrants (PMP-I) for mental health promotion among immigrants in the United States: a study protocol for a randomized controlled trial

Journal:	*BMJ Open*
Manuscript ID	bmjopen-2022-061353
Article Type:	Protocol
Date Submitted by the Author:	22-Jan-2022
Complete List of Authors:	Poudel-Tandukar, Kalpana ; University of Massachusetts Amherst, Nursing<br>Jacelon, Cynthia; University of Massachusetts Amherst<br>Martell, Christopher; University of Massachusetts Amherst<br>Poudel, Krishna C. ; University of Massachusetts Amherst, Public Health and Health Sciences<br>Rai, Shan; Bhutanese Christian Society of Western Massachusetts<br>Ramdam, Razu; Bhutanese Christian Society of Western Massachusetts<br>Laws, Holly; University of Massachusetts Amherst<br>Meyer, Jerrold; University of Massachusetts Amherst<br>Bertone-Johnson, E; University of Massachusetts Amherst<br>Hollon, Steven; Vanderbilt University
Keywords:	EPIDEMIOLOGY, MENTAL HEALTH, PSYCHIATRY, PREVENTIVE MEDICINE

SCHOLARONE™
Manuscripts

Peer-led family-centered Problem Management Plus for Immigrants (PMP-I) for mental health promotion among immigrants in the United States: a study protocol for a randomized controlled trial

Kalpana Poudel-Tandukar[1], Cynthia S Jacelon[1], Christopher Martell[2], Krishna C Poudel[3], Shan Rai[4], Razu Ramdam[4], Holly Laws[2], Jerrold S Meyer[2], Elizabeth R. Bertone-Johnson[5], Steven D Hollon[6]

[1]Elaine Marieb College of Nursing, University of Massachusetts Amherst, MA, USA

[2]Department of Psychological and Brain Sciences, College of Natural Sciences, University of Massachusetts Amherst, MA, USA

[3] Department of Health Promotion and Policy, School of Public Health and Health Sciences, University of Massachusetts Amherst, MA, USA

[4]Bhutanese Christian Society of Western Massachusetts, Springfield, MA, USA

[5]Department of Biostatistics and Epidemiology, School of Public Health and Health Sciences, University of Massachusetts Amherst, MA, USA

[6]Department of Psychology, Vanderbilt University, Nashville, TN, USA

*Correspondence to: Kalpana Poudel-Tandukar, Ph.D., MPH, MPHC, CGM

Associate Professor, Elaine Marieb College of Nursing, University of Massachusetts Amherst, 220 Skinner Hall, 651 North Pleasant St, Amherst, MA 01003-9299, USA

Tel: 1-413-545-5095; Fax: 1-413-577-2500

Email: kalpana@umass.edu

For peer review only - http://bmjopen.bmj.com/site/about/guidelines.xhtml

Abstract

Introduction

Research is needed to investigate potential preventive strategies to reduce stress and mental health burden and assess effective implementation among refugees and immigrants. Problem Management Plus (PMP) is a low-intensity multicomponent psychological intervention developed by the World Health Organization (WHO) that trained laypeople can deliver. PMP has been adapted as a prevention intervention and developed as Problem Management Plus for Immigrants (PMP-I), including psychoeducation, problem-solving, behavioral activation, and mind-body exercise, to address immigrants' multiple socio-cultural and emotional stressors. This trial aims to estimate the magnitude of the difference between PMP-I vs. community support services pamphlets on the primary outcomes of interest (stress, anxiety, and depressive symptoms) to inform the design of a large-scale intervention.

Methods and analysis

This trial will test preliminary effects of PMP-I vs. community support services pamphlets in a randomized controlled trial (N=116 families; 58 families for each condition intervention and control) on stress, anxiety, and depressive symptoms (primary outcomes), chronic physiological stress assessed in hair cortisol (secondary outcomes), and coping, family conflict resolution, and social networking (targets), with assessment at baseline, post-intervention, and 3-month post-intervention. Eligibility criteria for the primary study participants include Bhutanese adults 18 years or older resettled in Massachusetts with a score of 14 or below on the Patient Health Questionnaire (PHQ-9), a screening tool to exclude individuals with depressive symptoms (score

For peer review only - http://bmjopen.bmj.com/site/about/guidelines.xhtml

of 15 and more). All family members will be invited to participate in the family-based intervention (1-session/week for 5-week). Multilevel modeling will compare the longitudinal change in outcomes for each treatment arm while accounting for the clustering of participants within families.

Ethics and dissemination

The Institutional Review Board of the University of Massachusetts Amherst approved this study. The study results will be disseminated in peer-reviewed journal and conferences.

Trial registration number: NCT04453709

Prospectively registered on 1st July 2020. Recruitment of participants was delayed due to the COVID-19 pandemic and has started on August 17, 2021.

Keywords: Anxiety; Depression; Emotional Wellbeing; Mind-Body Intervention; Refugees; Social Wellbeing; Stress

Running title: Problem Management Plus for Immigrants in Bhutanese adults

For peer review only - http://bmjopen.bmj.com/site/about/guidelines.xhtml

Strengths and limitations of the study

- Whereas existing mental health interventions for immigrants are primarily based on treatment models to improve the access and quality of care for those with diagnosed mental health problems, this study is focused on developing, implementing, and pilot testing an effect of a culturally-tailored preventative behavioral intervention to reduce stress and prevent mental health problems among immigrants.
- This study includes culturally-tailored psychoeducation, behavioral activation, problem-solving, and mind-body interventions that help to address multiple psycho-socio-cultural stressors through revitalizing resources at the individual, family, and community levels.
- The proposed intervention will be delivered to participants in their family environment by interventionists from the same community they trust and understand their language and problems from their cultural lens.
- This study will be among the first to link a preventive intervention with both biomarkers of stress (hair cortisol) and perceived stress and, using longitudinal data, to examine change over time in stress.
- This study relies on self-report measures of anxiety and depressive symptoms though the clinical diagnosis is the gold standard. Such an approach is not feasible in community-based studies.

For peer review only

For peer review only - http://bmjopen.bmj.com/site/about/guidelines.xhtml

Introduction

Refugees resettled in the United States are vulnerable to mental health problems,[1, 2] such as anxiety and depression due to stress resulting from integrating into a new culture.[3-5] Refugees' risk for mental health problems increases during their acculturative process due to exposure to multiple stressors, such as adjustment to a new culture with limited language and socio-cultural skills, perceived discrimination, and a lack of culturally mediated and protective social support resources.[5, 6] Although mental health treatments are available to help alleviate the intrapersonal, social, and economic costs of mental disorders, refugees greatly underutilize these services.[1, 7, 8] Thus, evidence-based, culturally tailored preventative mental health interventions are needed for the growing number of refugees in the United States.

Existing interventions are focused explicitly on treatment models to provide quality care for those with diagnosed mental health problems[9] that do little to help reduce stress and prevent mental disorders for those who have not yet developed diagnosable symptoms. For prevention, a culturally tailored intervention that addresses multiple psycho-socio-cultural stressors, including social and cultural integration, holds good promise.[10, 11] Community-based preventative interventions that promote positive impacts of social and cultural behaviors on mental health outcomes by protective resources are needed for the growing number of refugees dealing with life complexities.[12, 13] A review of community-based mental health interventions in refugees resettled to the United States suggests[14] that counseling, health promotion, and skill-building workshops facilitated by refugee peers[15-17] are helpful to reduce the psychological distress of many refugees who may be struggling with individual or family difficulties. Specifically, the Centers for Disease Control and Prevention (CDC) recommends using a non-clinical, community support approach to prevent mental illness among refugees resettled in the United States.[18]

For peer review only - http://bmjopen.bmj.com/site/about/guidelines.xhtml

Problem Management Plus (PMP) is a low-intensity evidence-based psychological intervention developed by the World Health Organization (WHO) that trained laypeople can deliver.[19, 20] PMP systematically teaches four strategies: stress management through mind-body exercises,[21-24] problem-solving,[25] behavioral activation,[26-33] and skills to strengthen social support for individuals with psychological distress. PMP has been proven successful in reducing depression for women with mental disorders in Pakistan in a group setting.[34] We have adapted PMP to develop our Problem Management Plus for Immigrants (PMP-I) following a successful result of a pilot social and emotional wellbeing intervention. The pilot intervention included psychoeducation, mind-body exercise, problem-solving, and social support and reduced more than 50% prevalence of anxiety and depression from pre- to post-intervention among Bhutanese refugees when delivered in either a group[35] or a family setting.[36] While promising, these pilot results were drawn from only those receiving the treatment; no control group was available for comparison. Thus, the present study is to apply the adapted PMP in a randomized controlled trial. The present study is indicated for several reasons: our intervention model demands integration of social and emotional stressors; promising results of PMP in a non-controlled pilot study; the need to test the efficacy of PMP using the more rigorous randomized controlled trial (RCT) study design; strong evidence of family and community ties in health care process; and growing consensus among community, scientists, and policymakers on the need for family-based care models that are sustainable.

Objectives and hypothesis

The main objective of this study is to pilot test the feasibility and acceptability of PMP-I among Bhutanese adults 18 years or older living in Massachusetts with a score of 14 or below on the

For peer review only - http://bmjopen.bmj.com/site/about/guidelines.xhtml

Patient Health Questionnaire-9. Our central hypothesis is that PMP-I will reduce stress, anxiety, and depressive symptoms. We will test preliminary effects of PMP-I vs. community support services pamphlets in a randomized pilot trial (N=116 families; 58 families per intervention and control) on perceived stress,[37] anxiety and depressive symptoms (primary outcomes).[38] chronic physiological stress assessed in hair cortisol (secondary outcome), and self-efficacy,[39] coping,[40] family conflict resolution,[41] family satisfaction,[42] social support (targets),[43] and social networks[44] with assessments at baseline, post-, and 3-month post-intervention.

Methods

Design and setting

This study will be conducted among resettled Bhutanese adults living in Massachusetts. Since 2008, Bhutanese people have been resettled in various US states and are one of the largest groups of South Asian refugees (about 90, 000).[45] They bear a high burden of mental health problems both in the nation (depression: 20%; suicide rate: 21.5 per 100,000)[18] and in western Massachusetts (depression: 23.8%; anxiety: 34.5%).[46] Given the importance of family relationships, communication, and coping in mental health,[47] the preventative social and emotional wellbeing intervention was designed for resettled Bhutanese adults in western Massachusetts using a community-based participatory research (CBPR) approach.[35]

Study design: This mixed-methods study will incorporate a two-arm randomized controlled feasibility trial and qualitative evaluation of PMP-I intervention's acceptability to a range of stakeholders. The study protocol has been reported following the Standard Protocol Items:

For peer review only - http://bmjopen.bmj.com/site/about/guidelines.xhtml

Recommendations for Interventional Trials (SPIRIT). **Figure 1** shows the study flowchart, and **Figure 2** shows the SPIRIT figure.

Participant recruitment

Participant inclusion criteria using a screening measure

This study will include eligible parents and adult children aged 18 and above interested in participating as primary study participants. At baseline, we will use a screening tool to identify individuals without significant depressive symptoms, as we aim to evaluate the effect of our intervention to prevent depression rather than treat depression. Eligibility criteria for our primary study participants include Bhutanese adults 18 years or older (both parents and children of each family) resettled in Massachusetts with a score of 14 or below on the Patient Health Questionnaire (PHQ-9), a screening questionnaire for depression. Our statistical analysis will focus on data from primary study participants only with baseline PHQ-9 scores 14 or below. However, all other interested adult family members, both parents and their adult children, regardless of PHQ-9 score, will be invited to participate in the intervention. The PHQ-9 scores of participants will not be disclosed to anyone to maintain individual confidentiality. Besides, individuals with PHQ-9 screening score '15-19' (moderately severe depression) and '20-27' (severe depression) will be provided with feedback on their screening questionnaire outcomes confidentially. They will be encouraged to consult their primary health care providers.

Participant exclusion criteria

Participants with clinically diagnosed mental disorders and those taking psychiatric medications for any mental health problems will also be encouraged to participate in the family-based

For peer review only - http://bmjopen.bmj.com/site/about/guidelines.xhtml

intervention activities. However, in our primary statistical analysis, we will not consider data from those participants with PHQ-9 scores of 15 or above or diagnosed with mental health problems.

Informed consent

The principal investigator (PI) has prepared an informed consent document including an explanation on study background, screening, recruitment criteria, sample size, data collection, and intervention, study risks and benefits, confidentiality, National Institute of Mental Health Data Archive (NDA) data sharing policy, and hair samples collection procedure. Trained community research assistants (RAs) will inform screening and study procedures to each participant using UMass Amherst Institutional Review Board (IRB)-approved single informed consent form visiting in-person. Once participants understand study details, RAs will request their signature or initials or fingerprint for those who cannot write in the consent form before data collection. Participants will be reminded that their participation in the study is voluntary and free to leave the study without penalty.

Sample size and power calculations

The goal of the pilot project is to estimate the magnitude of the difference between the preventive intervention and the education control on the primary outcomes of interest to inform the design of a large-scale intervention. We conducted a power analysis to detect an effect size (ES) as small as ES=.30 with alpha = .05 and power of .80. We may find a larger effect in our pilot, but our understanding is that power estimates should be based on the smallest effect we want to detect rather than the size of the effect that we expect.[48] Analyses were performed using

For peer review only - http://bmjopen.bmj.com/site/about/guidelines.xhtml

Optimal Design[49] by accounting for the intra-correlation among family members of .10 and alpha = .05, we would have 80% power to detect a standardized difference of ES=.30 between two treatment groups of 116 families (58 per treatment arm) with an equal probability of being randomized to each of our two intervention arms.

Randomization

We will randomly allocate selected families into intervention and control groups using a random sampling method after the baseline survey. RAs are not aware of which group the family will be randomized to when collecting baseline data. Using a random number table, we will randomly assign 116 interested families (58 families per intervention and control). For random allocation, first, the PI will prepare the sampling frame that lists interested families, then assign a number to each family in the sampling frame, and finally select 116 numbers using a table of random numbers. We will assign a random number selected at the first attempt for intervention and the second attempt for control.

Procedures are in place for tracking the participants for intervention and follow-up (e.g., contact address and phone). RAs will visit selected families and brief them about study procedures, informed consent, and procedures to protect human subjects. Two adult members of selected families who meet the inclusion criteria and give informed consent will be recruited for the study. We will follow up with all families randomized to either study arm. We will not follow up with participants if they decide to end their participation at a particular time point of our study. But, we will include their already collected data in our analysis. Given our strong community networks and mobilization of community RAs, we anticipate low attrition rates in practice.

For peer review only - http://bmjopen.bmj.com/site/about/guidelines.xhtml

Problem Management Plus for Immigrants (PMP-I)

PMP-I is a 5-week, peer-led, culturally tailored mental health promotion program that includes psychoeducation, problem-solving, behavioral activation (90 minutes), breathing exercises, and yoga (90 minutes) in a family setting. PMP-I will use a structured approach, including once-a-week face-to-face sessions, yoga practice, breathing exercises, homework that includes practice activities, rebuilding individual skills, or learning new skills to reduce stress.

Our intervention aims to develop skills in coping adaptively in a new culture, seeking help and support for mental health problems, and other life skills opportunities that can improve their quality of life. **Module 1**: **Managing Stress** includes yoga, breathing exercise, stress-management sessions, and practice exercises to develop coping strategies that are most helpful to reduce stress and then plan a strategy to carry out those solutions. **Module 2: Managing Problems** includes practice exercises to identify the problems causing the most concern, develop solutions that are most helpful in addressing the problem, and then plan a strategy to carry out those solutions. **Module 3: Behavioral Activation** includes communication skill sessions and practice exercises to identify pleasant activities (time to yourself; connecting with others; self-care), breaking down the task into smaller steps, and schedule tasks, and then plan a strategy to carry out those tasks. **Module 4: Strengthening Social Support** includes social skills sessions and practice exercises to identify at least one person or service from whom the participant feels comfortable getting some support, and to plan exactly what the participant is going to do, and then schedule a day to carry out the tasks. **Module 5: Staying Well** includes practice exercises to make a plan that helps to create a supportive family environment.

For peer review only - http://bmjopen.bmj.com/site/about/guidelines.xhtml

Community interventionists (CIs) are trained community members with at least a high school level of education, and no formal training or prior experience with mental health will deliver the PMP-I. Dr. Christopher Martell, board-certified in cognitive and behavioral psychology and clinical psychology and a Massachusetts Licensed Psychologist, will provide 12 days of training to the interventionists in collaboration with the PI and Dr. Steven D. Hollon (Professor of Psychiatry, Psychology and Human Development) following the World Health Organization PMP Helpers' Training Guide[50] adapted for PMP-I. Classroom training includes information about stress, depression, mental health problems, the rationale for each intervention strategy, necessary helping skills, practice plan formulation, role-plays, peer observations, and group discussion related to core intervention concepts, practices, and supervision skills. Supervision involves discussing participants' progress and difficulties experienced when delivering strategies and role-playing on managing problems or practicing skills. They will use the PMP-I intervention manual to provide PMP-I to community members in family settings under field supervisors and PI's supervision. We will conduct a formal evaluation of the interventionists' readiness to implement/supervise the PMP-I intervention, such as using the manual, answering questions, managing time, using a fidelity checklist, practicing exercise, and role play to provide feedback as necessary.

A licensed yoga trainer will provide 4 hours of breathing exercises and 16 hours of yoga to CIs and field supervisors using a mind-body exercise training manual. Classroom training includes theoretical and practices to guide participants in mind-body exercises for attention to breath, body sensation, emotional awareness, and mental function on different postures of yoga practices such as *Pranayama* (3 poses) and *Asana* (21 poses). Training will include practice

For peer review only - http://bmjopen.bmj.com/site/about/guidelines.xhtml

assessment at the end to ensure that all field staff is trained, using a checklist, practice exercise, and role-plays.

Community Support Service Program

Bhutanese community members expressed that knowing the health and life skill development program available in their communities would benefit them in strengthening their life skills.[51] By considering their request, we have prepared pamphlets including names, contact, and service details of community and health organizations in the area where they live. CIs will distribute community support service program pamphlets to control families.

Primary outcome measures

Anxiety and Depressive Symptoms

The Hopkins Symptom Checklist-25 (HSCL-25) will be used to measure anxiety, and depressive symptoms experienced over the past month.[38] It is composed of a 10-item subscale for anxiety and a 15-item subscale for depression, with each item scored on a Likert scale from 1 (not at all) to 4 (extremely). The scale has high internal consistency (Cronbach's α) for anxiety (0.95) and depression (0.94) in the Bhutanese study.[35]

Perceived Stress

The 10-item Cohen Perceived Stress Scale (PSS) will be used to assess perceived stress.[37] The PSS uses a 5-point Likert scale (ranging from 0, "never" to 4, "very often") to assess psychological stress experienced during the past month, including the extent to which situations

For peer review only - http://bmjopen.bmj.com/site/about/guidelines.xhtml

felt unpredictable, uncomfortable, and overwhelming. In the Bhutanese study, the scale has high internal consistency (Cronbach's $\alpha = 0.80$).[35]

Secondary outcome measures

Physiological stress

We will use the enzyme-linked immunosorbent assay (ELISA) cortisol hair test (average hormone levels over the past three months) as a biomarker to measure physiological stress. Hair samples will be processed in the neuroendocrine lab at the University of Massachusetts Amherst.[52, 53] Sensitive and specific enzyme immunoassay (Arbor Assays) will be used for the analysis. The assay has intra- and inter-assay coefficients of variation of <10%.

Other measures

Coping strategy

Coping strategy will be measured using a 32-item Coping Strategies Inventory-Short Form (CSI-SF).[40] The CSI-SF includes two overall coping factors, Engagement and Disengagement, and four secondary factors, Problem Engagement, Problem Disengagement, Emotion Engagement, and Emotion Disengagement. The CSI-SF scale (Cronbach's $\alpha = 0.95$) has high internal consistencies in the Bhutanese study.[35] Participants were asked to rate their responses on a 5-point Likert-type scale ranging from *not at all (1)* to *very much (5)*.

Coping Self-efficacy

Self-efficacy will be measured using a 26-item Coping Self-efficacy (CSE) scale for coping with challenges and threats.[39] Each item of the scale will be rated on an 11-point scale Likert-type

For peer review only - http://bmjopen.bmj.com/site/about/guidelines.xhtml

scale ranging from *(0) cannot do at all, (5) moderately certain can do, and (10) certain can do*. The scale has high internal consistency (Cronbach's α = 0.96) in the previous Bhutanese study.[35]

Social support

Perceived social support will be measured using a 12-item Multidimensional Scale of Perceived Social Support (MSPSS),[43] including support from friends, family, and significant others. A sample item for this scale is, "My family tries to help me." Each item of the scale will be rated on a 5-point Likert-type scale ranging from *strongly disagree (1)* to *strongly agree (5)*. Graded items will be summed up to provide a total score, and higher scores indicate high social support. The scale has high internal consistency (Cronbach's α = 0.92) in the previous Bhutanese study.[35]

Social network

We will use a Lubben Social Network Scale-Revised (LSNS-R) to measure social networks among family and friendships.[44] It consists of six questions, which assess kinship ties, and a comparable set of six questions, which determine friend ties by replacing the word relatives with the word friends. We prepared three questions to measure cross-cultural social ties following a similar pattern. The scale has high internal consistency for kinship ties (Cronbach's α = 0.78), friendship ties (Cronbach's α = 0.80), and cross-cultural social ties (Cronbach's α = 0.74) in the previous Bhutanese study.[35] These items will be scored on a five-point Likert scale ranging from *none (0)* to *9 or more or always (5)*.

Family conflict resolution

For peer review only - http://bmjopen.bmj.com/site/about/guidelines.xhtml

Family conflict resolution, including positive or negative resolution, effective communication, and discussion of differences, will be measured using a 17-item version of the "Family Conflict Resolution" scale.[41] This scale has high internal consistency (Cronbach's $\alpha = 0.90$) in the previous Bhutanese study.[35] Participants will be asked to respond on a 7-point Likert-type scale, ranging from *never (1)* to *always (7)*.

Family satisfaction: Family satisfaction with various aspects of family functioning, including family closeness, flexibility, and communication, will be measured using a 10-item family satisfaction scale.[54] Participants will be asked to respond on a 5-point Likert-type scale, ranging from *very dissatisfied (1)* to *extremely satisfied (5)*.

Process evaluation

The PMP trainer's training guidelines provide specific tools for evaluating and monitoring the intervention, which we use to monitor intervention delivery fidelity. These tools are PMP Quiz, PMP Helper's Supervision Form, PMP Helper Classroom-based Competency Assessment, PMP Helper In-field based Competency Assessment, PMP Trainer/Supervisor Competency Assessment, and Session-by-Session Checklists for PMP Helpers.[50] We have adapted these tools in the context of our program contents. Using these standard tools, we will evaluate session-by-session classroom and in-field based competencies of community interventionists and field supervisors and provide them feedback as needed using supervision forms, role-plays, group discussion, and training.

At the field level, field supervisors will monitor intervention sessions delivered by community interventionists using standard checklists. Items include adherence to the manual,

For peer review only - http://bmjopen.bmj.com/site/about/guidelines.xhtml

percent of intervention content administered, proper use of time/materials, and adequate response to participants' questions. They will also monitor participants' engagement, acceptability, and satisfaction via brief questionnaires with participants and interventionists during and after intervention completion. Moreover, community interventionists will be asked to complete a structured checklist on the attendance, compliance, and satisfaction towards intervention components immediately after each session.

The PI will conduct a focus group discussion (FGD) in the Nepali language with interventionists, supervisors, and participants separately to collect information on barriers and facilitators of intervention, perceptions about whether the intervention met participants' needs, and feedback on how effectively the program team worked with participants. Interviews and FGD will be documented verbatim in a written transcript for subsequent analysis. All qualitative data will be analyzed using thematic content analysis.[55] Feedback provided by the field staff will be reviewed and coded to identify recurrent themes regarding the intervention's acceptability. Fidelity data will be used to assess intervention content and transmission.

Data management

All interviews will be conducted with the utmost privacy and confidentiality. Each interested and eligible adult participant in the family will be interviewed individually, in a private place where they feel comfortable, by our trained community RAs. The RAs will ensure audio and visual privacy at these sites, and ensure data confidentiality. RAs will reassure participants that numerical codes would be used in place of names in all records to ensure confidentiality. The survey materials (questionnaires, transcriptions, and field notes) will be stored in a locked cabinet in the PI's office. Data entry will be done on the PI's office computer (encrypted and password protected) under the full supervision of the PI. The original data will be kept on

For peer review only - http://bmjopen.bmj.com/site/about/guidelines.xhtml

OneDrive, a secure, networked university data storage system. De-identified data sets will be used for statistical analyses. The PI herself will do data analysis and documentation. All information will be presented in aggregate form in the manuscript or conference abstract, and no individual respondent will be identified.

Data analysis

We will compare baseline characteristics of intervention and control groups using chi-square and t-tests as appropriate. While differences between groups are not expected because of the randomization used in the study design, variables showing significant differences between the two groups will be included as covariates in primary analyses. The primary analyses will test whether participants' outcomes in the PMP-I arm differ from those in the control arm. Multilevel modeling will compare outcomes of each treatment arm while accounting for the clustering of participants within families. Continuous outcomes will be analyzed using hierarchical linear modeling, and dichotomous outcomes will be analyzed using multilevel generalized linear models with a Bernoulli distribution appropriate to nonlinear binary outcomes.[56]

We expect approximately 2 to 4 members for each of the 58 families in each treatment arm, and the correlation among family members' responses will be accounted for in the model. Hierarchical or multilevel modeling is suited to these data as it accounts for the clustering of members within families and unbalanced designs (i.e., different family sizes).[56] This will be an intention-to-treat type of analysis, as multilevel modeling allows retention of all participants irrespective of the number of sessions attended (multilevel modeling uses maximum likelihood estimation, one of the recommended ways of handling missing data). The analysis will estimate endpoint outcomes based on repeated measures (Level 1) within individuals (Level 2) within

For peer review only - http://bmjopen.bmj.com/site/about/guidelines.xhtml

families (Level 3). Separate models will be created to evaluate the relationships between mediators (targets) and outcomes and explore mediators (e.g., coping) of intervention-outcome relation. All analyses will be performed using SAS, version 9 (SAS Institute Inc, Cary, NC).

Independent Safety Monitor

We will select an Independent Safety Monitor (ISM) with mental health expertise, whose primary responsibility is to provide independent monitoring of this clinical trial in a timely fashion. Overall, the ISM will review enrollment data, safety data, and data integrity to maintain safety in the trial. The PI will submit data reports once a year to the ISM. The report will include the key variables necessary for monitoring the safety and quality of data collection and the integrity of the study, including inclusion criteria, informed consent, subject enrollment and retention, data confidentiality, intervention compliance, drop outs, adverse events, protocol compliance, data quality, and baseline characteristics of study participants. The ISM will have access to all safety and data quality information collected and will have the authority to stop the study if it is determined that there are unacceptable risks to participants. The ISM also will review the study protocol, informed consent, and all relevant documents before the onset of the study, and will review and approve amendments to these documents. The ISM will issue a monitoring report to the PI following each review. The PI will submit all review reports to the UMass Amherst IRB and NIMH Program Officer in annual progress reports.

Trial management

The PI will assume overall responsibility of trial management working together with the entire research team throughout the project, meeting monthly with Co-Investigators (psychiatrist,

For peer review only - http://bmjopen.bmj.com/site/about/guidelines.xhtml

cognitive behavior therapist and epidemiologist) and once every week with field staff (supervisors, interventionists, and research assistants) via in-person or zoom or text message as needed. During the trial, experienced field supervisors from the Bhutanese community, who are trained as a community health worker and have worked with the PI in previous family-based mental health intervention studies with depressive and suicidal ideation outcomes, will take responsibility for the day-to-day oversight of the participants and field teams in the implementation of the trial. Field supervisors will immediately report any noted adverse events among participants to the PI. The PI will report adverse events data to the Independent Safety Monitor (ISM), UMass Amherst IRB, and National Institute of Mental Health (NIMH) Program Officer following NIMH guidelines for reportable events, as described below.

Adverse events reporting

Throughout the study period, all study participants will be monitored daily by the field supervisors under the PI's supervision. Field supervisors will request study participants and their family members to immediately report any unanticipated serious adverse events in their family, such as 1) reporting suicidal ideation or attempts, hospitalization, disability, and/or death; 2) discomfort with the PMP-I program content and/or evaluation procedures, and 3) risk of a breach of confidentiality, of the collected data and/or by program personnel to field supervisor or PI directly. Field supervisors will immediately report details of such adverse events to PI. The PI will be responsible for reporting them to the UMass Amherst IRB, ISM, and NIMH Program Officer by secure email within ten business days of the study team becoming aware of any serious adverse events. The PI will be responsible for summarizing all adverse events that are

For peer review only - http://bmjopen.bmj.com/site/about/guidelines.xhtml

deemed expected and/or unrelated to the study in the annual progress report submitted to the UMass Amherst IRB, ISM, and NIMH Program Officer by secure email.

Discussion

This study reports an RCT protocol that tests PMP-I's feasibility, acceptability, and preliminary outcomes with trained community facilitators. This study is built on prior research that has shown the effectiveness of social and emotional wellbeing intervention, including psychoeducation, problem-solving, social support, and mind-body exercises, to reduce stress, anxiety, and depressive symptoms among Bhutanese adults resettled in MA at a group[35] and family settings[36] using a pre-and post-test intervention design. Our project designed for Bhutanese immigrants includes evidence-based interventions of specific relevance to this community, such as psychoeducation,[57] problem solving,[25] behavioral activation,[26-33] mind-body exercises,[21-24] and strengthening social support to address identified social (e.g., social isolation, language difficulties) and emotional (e.g., lack of self-esteem or self-efficacy)[51] stressors by strengthening protective factors (e.g., resilience or coping).[58, 59] This study is innovative as it will be the first culturally tailored, preventive, family-based, multi-component behavioral intervention driven by the community to reduce stress. We will have pilot-tested a preventative mental health intervention for Bhutanese adults upon completion. This study can be expected to impact reducing stress and promoting immigrants' mental wellbeing.

Our project is likely to be replicated with other immigrant communities with minimal adaptation over the long term for three reasons. First, our intervention is guided by a strengths-based approach in which we plan to include community strengths. This principle can be applied to capture and integrate the strengths of any community. Second, our program prioritizes the

For peer review only - http://bmjopen.bmj.com/site/about/guidelines.xhtml

training of community members as interventionists, as these are individuals whom the community trusts, who share the same cultural lens as the community and thus can well understand language and specific challenges, and who have a vested interest in the strength and resilience of their community. This aspect of our intervention design is easily adaptable to other populations. Finally, intervention is designed to be delivered in family settings where participants are most comfortable, and family members can support each other throughout their lives. This component is crucial in collectivistic societies where family bonds and group identity are strong. Thus, our family-based strategies could be replicable in other immigrant groups, where there are similarities in social and emotional stressors, challenges, community strengths (coping, resilience, social support), strong family support, and cultural preference of native community counselors for their mental health consultation. Our strength-based and peer-led strategies promote community engagement and make the intervention sustainable.[60-62]

Patient and public involvement

Patients or the public were not involved in the design, or conduct, or reporting, or dissemination plans of our research.

Trial status

At the time of manuscript submission, trial was ongoing. Results of this study are expected in mid 2024.

For peer review only - http://bmjopen.bmj.com/site/about/guidelines.xhtml

List of abbreviations

CBPR: Community-Based Participatory Research; CDC: Centers for Disease Control and Prevention; CIs: Community Interventionists; CSE: Coping Self-efficacy; CSI-SF: Coping Strategies Inventory-Short Form; ELISA: Enzyme-Linked Immunosorbent Assay; ES: Effect Size; FGD: Focus Group Discussion; HSCL-25: Hopkins Symptom Checklist-25; IRB: Institutional Review Board; ISM: Independent Safety Monitor; LSNS-R: Lubben Social Network Scale-Revised; MSPSS: Multidimensional Scale of Perceived Social Support; NDA: National Institute of Mental Health Data Archive; NIMH: National Institute of Mental Health; PHQ-9: Patient Health Questionnaire; PI: Principal Investigator; PMP: Problem Management Plus; PMP-I: Problem Management Plus for Immigrants; PSS: Perceived Stress Scale; RAs: Research Assistants; RCT: Randomized Controlled Trial; SPIRIT: Standard Protocol Items: Recommendations for Interventional Trials; WHO: World Health Organization

For peer review only

For peer review only - http://bmjopen.bmj.com/site/about/guidelines.xhtml

Declarations

Ethics and dissemination

The Institutional Review Board of the University of Massachusetts Amherst approved this study and certified that it was performed according to the ethical standards laid down in the 1964 Declaration of Helsinki and its later amendments. All participants enroll in the study will provide written informed consent. The results of this study will be disseminated through peer-reviewed scienfic journals and conferences.

Consent for publication

Not applicable

Availability of data and materials

At the time of manuscript submission, trial was ongoing. Results of this study are expected mid 2024. The PI will have access to the final data trial set.

Competing interests

The author(s) declared no potential conflicts of interest with respect to the research, authorship, and/or publication of this article.

Funding

This trial is funded by the National Institute of Mental Health of the National Institutes of Health under Award Number R34MH118396 (PI: Dr. Kalpana Poudel-Tandukar). The content is solely

For peer review only - http://bmjopen.bmj.com/site/about/guidelines.xhtml

the authors' responsibility and does not necessarily represent the official views of the National Institutes of Health.

Authors' contributions

KPT conceived the study and drafted the study and trial protocols. All authors were involved in the design of the study; KPT, CSJ, CM, KCP, SR, RR, HL, JSM, ERBJ, and SDH were involved in revising the study protocol for ethics review, and all authors were involved in commenting on and revising the trial protocol. All authors read and approved the final manuscript.

Acknowledgments

Not applicable

Authors' information

[1]Elaine Marieb College of Nursing, University of Massachusetts Amherst, MA, USA

[2]Department of Psychological and Brain Sciences, College of Natural Sciences, University of Massachusetts Amherst, MA, USA

[3] Department of Health Promotion and Policy, School of Public Health and Health Sciences, University of Massachusetts Amherst, MA, USA

[4]Bhutanese Christian Society of Western Massachusetts, Springfield, MA, USA

[5]Department of Biostatistics and Epidemiology, School of Public Health and Health Sciences, University of Massachusetts Amherst, MA, USA

[6]Department of Psychology, Vanderbilt University, Nashville, TN, USA

For peer review only - http://bmjopen.bmj.com/site/about/guidelines.xhtml

References

1. Taylor EM, Yanni EA, Pezzi C, Guterbock M, Rothney E, Harton E, Montour J, Elias C, Burke H. Physical and mental health status of Iraqi refugees resettled in the United States. J Immigr Minor Health 2014;16:1130-7.

2. Wagner J, Burke G, Kuoch T, Scully M, Armeli S, Rajan TV. Trauma, healthcare access, and health outcomes among Southeast Asian refugees in Connecticut. J Immigr Minor Health 2013;15:1065-72.

3. Alemi Q, Stempel C. Discrimination and distress among Afghan refugees in northern California: The moderating role of pre- and post-migration factors. PLoS One 2018;13:e0196822.

4. Ellis BH, Lankau EW, Ao T, Benson MA, Miller AB, Shetty S, Lopes Cardozo B, Geltman PL, Cochran J. Understanding Bhutanese refugee suicide through the interpersonal-psychological theory of suicidal behavior. Am J Orthopsychiatry 2015;85:43-55.

5. Vonnahme LA, Lankau EW, Ao T, Shetty S, Cardozo BL. Factors Associated with Symptoms of Depression Among Bhutanese Refugees in the United States. J Immigr Minor Health 2015;17:1705-14.

6. Williams ME, Thompson SC. The use of community-based interventions in reducing morbidity from the psychological impact of conflict-related trauma among refugee populations: a systematic review of the literature. J Immigr Minor Health 2011;13:780-94.

7. Ellis BH, Lincoln AK, Charney ME, Ford-Paz R, Benson M, Strunin L. Mental health service utilization of Somali adolescents: religion, community, and school as gateways to healing. Transcult Psychiatry 2010;47:789-811.

For peer review only - http://bmjopen.bmj.com/site/about/guidelines.xhtml

8. Colucci E, Szware J, Minas H, Paxton G, Guerra C. The utilization of mental health services by children and young people from a refugee background: A systematic literature review. International Journal of Culture and Mental Health 2012;7:86-108.

9. Swinson Evans T, Berkman N, Brown C, Gaynes B, Palmieri Weber R. Disparities within serious mental illness. Technical Brief No. 25. (Prepared by the RTI International–University of North Carolina Evidence-based Practice Center under Contract No. 290-2015-00011-I). AHRQ Publication No.16-EHC027-EF. Rockville, MD: Agency for Healthcare Research and Quality; May 2016. , 2016.

10. Goodkind JR, Hess JM, Isakson B, LaNoue M, Githinji A, Roche N, Vadnais K, Parker DP. Reducing refugee mental health disparities: a community-based intervention to address postmigration stressors with African adults. Psychol Serv 2014;11:333-46.

11. Weine SM. Developing preventive mental health interventions for refugee families in resettlement. Fam Process 2011;50:410-30.

12. Kirmayer LJ, Narasiah L, Munoz M, Rashid M, Ryder AG, Guzder J, Hassan G, Rousseau C, Pottie K. Common mental health problems in immigrants and refugees: general approach in primary care. CMAJ 2011;183:E959-67.

13. Pumariega AJ, Rothe E, Pumariega JB. Mental health of immigrants and refugees. Community Ment Health J 2005;41:581-97.

14. Peterson C, Poudel-Tandukar K, Sanger K, Jacelon CS. Improving Mental Health in Refugee Populations: A Review of Intervention Studies Conducted in the United States. Issues Ment Health Nurs 2020;41:271-82.

For peer review only

For peer review only - http://bmjopen.bmj.com/site/about/guidelines.xhtml

15. Im H, Rosenberg R. Building Social Capital Through a Peer-Led Community Health Workshop: A Pilot with the Bhutanese Refugee Community. J Community Health 2016;41:509-17.

16. Birman D, Beehler S, Harris EM, Everson ML, Batia K, Liautaud J, Frazier S, Atkins M, Blanton S, Buwalda J, Fogg L, Cappella E. International Family, Adult, and Child Enhancement Services (FACES): a community-based comprehensive services model for refugee children in resettlement. Am J Orthopsychiatry 2008;78:121-32.

17. Boehnlein JK, Kinzie JD, Sekiya U, Riley C, Pou K, Rosborough B. A ten-year treatment outcome study of traumatized Cambodian refugees. J Nerv Ment Dis 2004;192:658-63.

18. CDC. Suicide and suicidal ideation among Bhutanese refugees--United States, 2009-2012. MMWR Morb Mortal Wkly Rep 2013;62:533-6.

19. Dawson KS, Bryant RA, Harper M, Kuowei Tay A, Rahman A, Schafer A, van Ommeren M. Problem Management Plus (PM+): a WHO transdiagnostic psychological intervention for common mental health problems. World Psychiatry 2015;14:354-7.

20. World Health Organization. Problem Management Plus (PM+): Individual psychological help for adults impaired by distress in communities exposed to adversity. (Generic field-trial version 1.0). Geneva, WHO, 2016.

21. Wahbeh H, Elsas SM, Oken BS. Mind-body interventions: applications in neurology. Neurology 2008;70:2321-8.

22. Falsafi N. A Randomized Controlled Trial of Mindfulness Versus Yoga: Effects on Depression and/or Anxiety in College Students. J Am Psychiatr Nurses Assoc 2016;22:483-97.

For peer review only

For peer review only - http://bmjopen.bmj.com/site/about/guidelines.xhtml

23. Prathikanti S, Rivera R, Cochran A, Tungol JG, Fayazmanesh N, Weinmann E. Treating major depression with yoga: A prospective, randomized, controlled pilot trial. PLoS One 2017;12:e0173869.

24. de Manincor M, Bensoussan A, Smith C, Fahey P, Bourchier S. Establishing key components of yoga interventions for reducing depression and anxiety, and improving well-being: a Delphi method study. BMC Complement Altern Med 2015;15:85.

25. Cuijpers P, de Wit L, Kleiboer A, Karyotaki E, Ebert DD. Problem-solving therapy for adult depression: An updated meta-analysis. Eur Psychiatry 2018;48:27-37.

26. Martell CR, Dimidjian S, Hermann-Dunn R. Behavioral activation for depression: a clinician's guide. Gullford New York, NY.; 2010.

27. Mazzucchelli T, R. K, Rees C. Behavioral activation treatments for depression in adults: a meta-analysis and review. . Clin Psychol Sci Pract 2009;16:383-411.

28. Kanter JW, Manos RC, Bowe WM, Baruch DE, Busch AM, Rusch LC. What is behavioral activation? A review of the empirical literature. Clin Psychol Rev 2010;30:608-20.

29. Cuijpers P, van Straten A, Warmerdam L. Behavioral activation treatments of depression: a meta-analysis. Clin Psychol Rev 2007;27:318-26.

30. Cuijpers P, van Straten A, Warmerdam L. Problem solving therapies for depression: a meta-analysis. Eur Psychiatry 2007;22:9-15.

31. Kanter JW, Bush AM, Rusch LC. Behavioural activation: the CBT distinctive feature series. New York: Routledge.; 2009.

32. Veale D. Behavioural activation for depression. Adv Psychiatr Treat 2008;14:29-36.

33. Chowdhary N, Anand A, Dimidjian S, Shinde S, Weobong B, Balaji M, Hollon SD, Rahman A, Wilson GT, Verdeli H, Araya R, King M, Jordans MJ, Fairburn C, Kirkwood B,

For peer review only

For peer review only - http://bmjopen.bmj.com/site/about/guidelines.xhtml

Patel V. The Healthy Activity Program lay counsellor delivered treatment for severe depression in India: systematic development and randomised evaluation. Br J Psychiatry 2015;208:381-8.

34. Khan MN, Hamdani SU, Chiumento A, Dawson K, Bryant RA, Sijbrandij M, Nazir H, Akhtar P, Masood A, Wang D, Wang E, Uddin I, Ommeren MV, Rahman A. Evaluating feasibility and acceptability of a group WHO trans-diagnostic intervention for women with common mental disorders in rural Pakistan: a cluster randomised controlled feasibility trial. Epidemiol Psychiatr Sci 2017:1-11.

35. Poudel-Tandukar K, Jacelon CS, Rai S, Ramdam P, Bertone-Johnson ER, Hollon SD. Social and Emotional Wellbeing (SEW) Intervention for Mental Health Promotion Among Resettled Bhutanese Adults in Massachusetts. Community Ment Health J 2021;57:1318-27.

36. Poudel-Tandukar K, Jacelon CS, Poudel KC, Bertone-Johnson ER, Rai S, Ramdam P, Hollon SD. Mental health promotion among resettled Bhutanese adults in Massachusetts: Results of a peer-led family-centred Social and Emotional Well-being (SEW) intervention study. Health Soc Care Community 2021.

37. Cohen S, Kamarck T, Mermelstein R. A global measure of perceived stress. J Health Soc Behav 1983;24:385-96.

38. Derogatis LR, Lipman RS, Rickels K, Uhlenhuth EH, Covi L. The Hopkins Symptom Checklist (HSCL): a self-report symptom inventory. Behav Sci 1974;19:1-15.

39. Chesney MA, Neilands TB, Chambers DB, Taylor JM, Folkman S. A validity and reliability study of the coping self-efficacy scale. Br J Health Psychol 2006;11:421-37.

40. Tobin DL. Coping strategies therapy for bulimia nervosa. Washington, DC: American Psychological Association Press, 2000., 2000.

For peer review only - http://bmjopen.bmj.com/site/about/guidelines.xhtml

41. Roskos PT, Handal PJ, Ubinger ME. Family Conflict Resolution: Its Measurement and Relationship with Family Conflict and Psychological Adjustment. Psychology 2010;1:370-76.

42. Olson DH. Family Satisfaction Scale. Minneapolis, MN: Life Innovations, Inc.; 2010.

43. Zimet GD, Dahlem NW, Zimet SG, Farley GK. The multidimensional scale of perceived social support. Journal of Personality Assessment 1988;52:30-41.

44. Lubben J, Blozik E, Gillmann G, Iliffe S, von Renteln Kruse W, Beck JC, Stuck AE. Performance of an abbreviated version of the Lubben Social Network Scale among three European community-dwelling older adult populations. Gerontologist 2006;46:503-13.

45. United States Embassy Kathmandu. U.S. Ambassador Bids Farewell to 90,000th Refugee to Resettle to the United States. 21 September, 2016. Accessed July 24, 2017 From: https://np.usembassy.gov/u-s-ambassador-bids-farewell-90000th-refugee-resettle-united-states/

2016.

46. Poudel-Tandukar K, Bertone-Johnson ER, KC P, Poudel KC. Family-engaged preventative behavioral interventions are needed to promote mental health among HIV-infected individuals living in resource-poor settings. American Public Health Association Annual Meeting and Exposition (APHA), Philadelphia, USA. November 2-6, 2019. (Abstract Number: 438575): Oral Presentation. 2019.

47. Slobodin O, de Jong JT. Family interventions in traumatized immigrants and refugees: A systematic review. Transcult Psychiatry 2015;52:723-42.

48. Kraemer HC, Mintz J, Noda A, Tinklenberg J, Yesavage JA. Caution regarding the use of pilot studies to guide power calculations for study proposals. Arch Gen Psychiatry 2006;63:484-9.

For peer review only - http://bmjopen.bmj.com/site/about/guidelines.xhtml

49. Raudenbush, S. W., et al. (2011). Optimal Design Software for Multi-level and Longitudinal Research (Version 3.01) [Software]. Available from www.wtgrantfoundation.org.

50. World Health Organization. Problem Management Plus (PM+) Training Guide for PM+ Helpers: To accompany the PM+ intervention manual: Problem Management Plus: Individual psychological help for adults impaired by distress in communities exposed to adversity –Field Version 1.0 WHO (2016). Series on Low-Intensity Psychological Interventions – 2. Geneva, WHO, 2018. .

51. Poudel-Tandukar K, Jacelon CS, Chandler GE, Gautam B, Palmer PH. Sociocultural Perceptions and Enablers to Seeking Mental Health Support Among Bhutanese Refugees in Western Massachusetts. Int Q Community Health Educ 2019;39:135-45.

52. Meyer J, Novak M, Hamel A, Rosenberg K. Extraction and analysis of cortisol from human and monkey hair. J Vis Exp 2014:e50882.

53. Meyer JS, Novak MA. Minireview: Hair cortisol: a novel biomarker of hypothalamic-pituitary-adrenocortical activity. Endocrinology 2012;153:4120-7.

54. Olson DH, Wilson M. Family Satisfaction. In Olson, D.H., and colleagues. Families: What Makes Them Work. Newbury Park, CA: Sage Publishing.; 1982, 1989.

55. Green J, Thorogood N. Qualitative methods for health research. Thousands Oaks, CA, USA: SAGE Publishing; 2014. 2014.

56. Raudenbush S, W., Bryk. AS. Hierarchical Linear Models: Applications and Data Analysis Methods. 2nd ed. Thousand Oaks: Sage Publications. 2002.

57. Zlotnick C, Johnson SL, Miller IW, Pearlstein T, Howard M. Postpartum depression in women receiving public assistance: pilot study of an interpersonal-therapy-oriented group intervention. Am J Psychiatry 2001;158:638-40.

For peer review only - http://bmjopen.bmj.com/site/about/guidelines.xhtml

58. Poudel-Tandukar K, Jacelon CS, Bertone-Johnson ER, Gautam B, Palmer PH, Hollon SD. Coping strategies and stress among resettled Bhutanese adults in Massachusetts. Am J Orthopsychiatry 2020;90:502-09.

59. Poudel-Tandukar K, Chandler GE, Jacelon CS, Gautam B, Bertone-Johnson ER, Hollon SD. Resilience and anxiety or depression among resettled Bhutanese adults in the United States. Int J Soc Psychiatry 2019:20764019862312.

60. Singla DR, Kohrt BA, Murray LK, Anand A, Chorpita BF, Patel V. Psychological Treatments for the World: Lessons from Low- and Middle-Income Countries. Annu Rev Clin Psychol 2017;13:149-81.

61. Webel AR, Okonsky J, Trompeta J, Holzemer WL. A systematic review of the effectiveness of peer-based interventions on health-related behaviors in adults. Am J Public Health 2010;100:247-53.

62. Petosa R, Smith LH. Peer mentoring for health behaviors change: A systematic review. . American Journal of Health Education 2014;45:351-57.

For peer review only

For peer review only - http://bmjopen.bmj.com/site/about/guidelines.xhtml

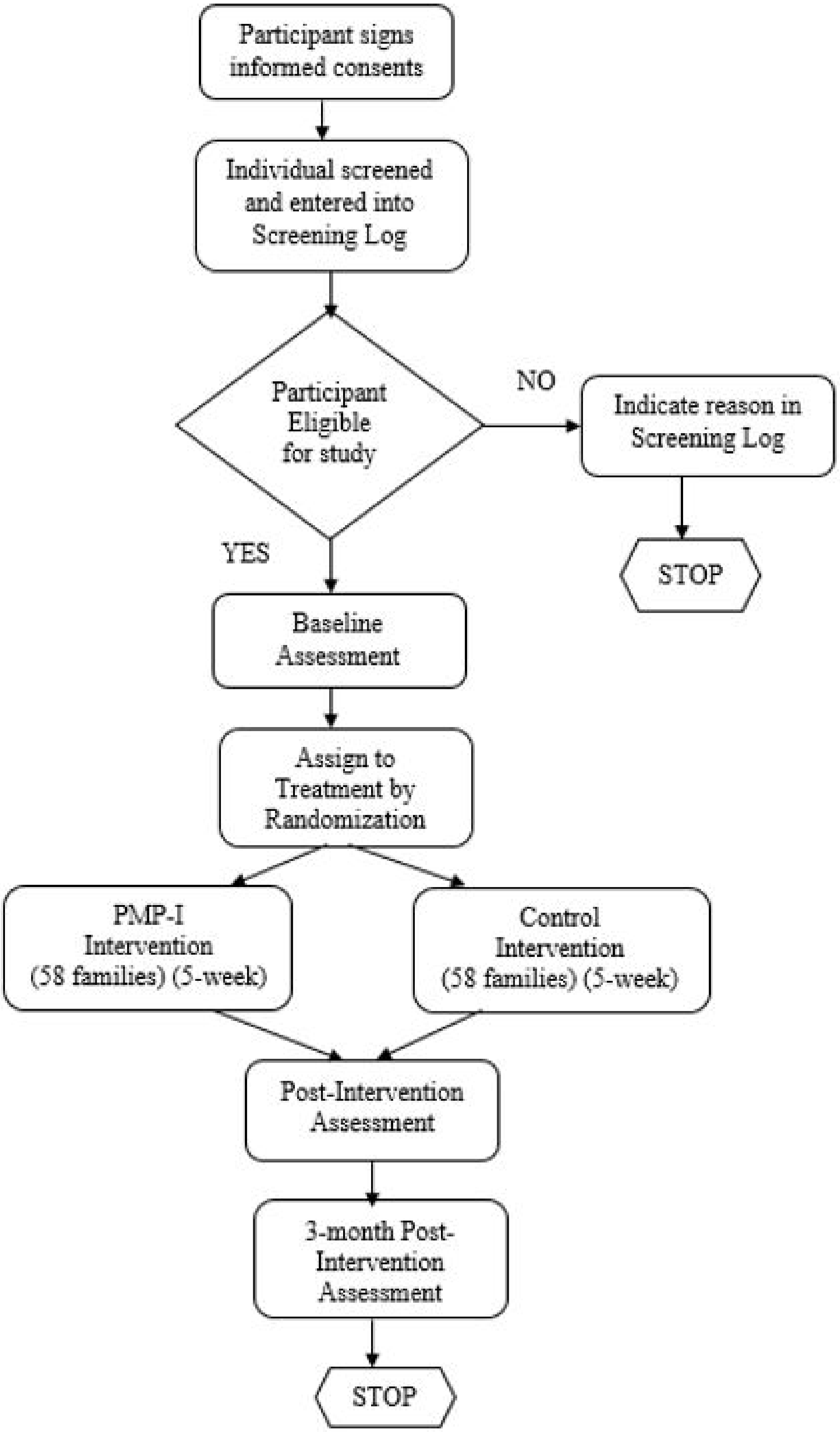

Figure 1 Flowchart of the study

For peer review only - http://bmjopen.bmj.com/site/about/guidelines.xhtml

Assessment	***Screening Visit 1***	***Baseline, Enrollment Randomization: Visit 1***	***Intervention Session 1 Visit 2***	***Intervention Session 2 Visit 3***	***Intervention Session 3 Visit 4***	***Intervention Session 4 Visit 5***	***Intervention Session 5 Visit 6***	***Post-intervention Visit 7***	***3-month Follow-up Final Visit***
Informed Consent Form	*X*								
Screening tool	*X*								
Inclusion/Exclusion Criteria	*X*								
Demographics		*X*						*X*	*X*
Blood pressure		*X*						*X*	*X*
Bodyweight & height		*X*						*X*	*X*
Waist circumference		*X*						*X*	*X*
Hair samples		*X*							*X*
Stress, Anxiety & Depression		*X*						*X*	*X*
Family & Social Support		*X*						*X*	*X*
Coping Strategies		*X*						*X*	*X*
Self-efficacy		*X*						*X*	*X*
Family Conflict Resolution		*X*						*X*	*X*
Family Satisfaction		*X*							
Enrollment/Randomization		*X*							
Intervention Session and its assessment using fidelity form			*X*	*X*	*X*	*X*	*X*		
Adverse Events		*X*	*X*	*X*	*X*	*X*	*X*	*X*	*X*

Figure 2 Overview of the study measure

For peer review only

SPIRIT
STANDARD PROTOCOL ITEMS: RECOMMENDATIONS FOR INTERVENTIONAL TRIALS

SPIRIT 2013 Checklist: Recommended items to address in a clinical trial protocol and related documents*

Section/item	Item No	Description	Addressed on page number
**Administrative information**			
Title	1	Descriptive title identifying the study design, population, interventions, and, if applicable, trial acronym	_____1________
Trial registration	2a	Trial identifier and registry name. If not yet registered, name of intended registry	_____3_______
	2b	All items from the World Health Organization Trial Registration Data Set	_____NA______
Protocol version	3	Date and version identifier	_____NA_____
Funding	4	Sources and types of financial, material, and other support	____24-25______
Roles and responsibilities	5a	Names, affiliations, and roles of protocol contributors	____25_______
	5b	Name and contact information for the trial sponsor	____24-25______
	5c	Role of study sponsor and funders, if any, in study design; collection, management, analysis, and interpretation of data; writing of the report; and the decision to submit the report for publication, including whether they will have ultimate authority over any of these activities	____24-25______
	5d	Composition, roles, and responsibilities of the coordinating centre, steering committee, endpoint adjudication committee, data management team, and other individuals or groups overseeing the trial, if applicable (see Item 21a for data monitoring committee)	___ 19_________

For peer review only - http://bmjopen.bmj.com/site/about/guidelines.xhtml

Introduction

Background and rationale	6a	Description of research question and justification for undertaking the trial, including summary of relevant studies (published and unpublished) examining benefits and harms for each intervention	___5 - 7_______
	6b	Explanation for choice of comparators	____13_________
Objectives	7	Specific objectives or hypotheses	____6 - 7_______
Trial design	8	Description of trial design including type of trial (eg, parallel group, crossover, factorial, single group), allocation ratio, and framework (eg, superiority, equivalence, noninferiority, exploratory)	____7 - 8______

Methods: Participants, interventions, and outcomes

Study setting	9	Description of study settings (eg, community clinic, academic hospital) and list of countries where data will be collected. Reference to where list of study sites can be obtained	____ 7_________
Eligibility criteria	10	Inclusion and exclusion criteria for participants. If applicable, eligibility criteria for study centres and individuals who will perform the interventions (eg, surgeons, psychotherapists)	____8 - 9_______
Interventions	11a	Interventions for each group with sufficient detail to allow replication, including how and when they will be administered	____11 - 13_____
	11b	Criteria for discontinuing or modifying allocated interventions for a given trial participant (eg, drug dose change in response to harms, participant request, or improving/worsening disease)	___ 19 - 21______
	11c	Strategies to improve adherence to intervention protocols, and any procedures for monitoring adherence (eg, drug tablet return, laboratory tests)	___ 19 - 21______
	11d	Relevant concomitant care and interventions that are permitted or prohibited during the trial	____NA________
Outcomes	12	Primary, secondary, and other outcomes, including the specific measurement variable (eg, systolic blood pressure), analysis metric (eg, change from baseline, final value, time to event), method of aggregation (eg, median, proportion), and time point for each outcome. Explanation of the clinical relevance of chosen efficacy and harm outcomes is strongly recommended	___13 - 16______
Participant timeline	13	Time schedule of enrolment, interventions (including any run-ins and washouts), assessments, and visits for participants. A schematic diagram is highly recommended (see Figure)	___ 34_________

For peer review only - http://bmjopen.bmj.com/site/about/guidelines.xhtml

Sample size	14	Estimated number of participants needed to achieve study objectives and how it was determined, including clinical and statistical assumptions supporting any sample size calculations	___9 - 10_______
Recruitment	15	Strategies for achieving adequate participant enrolment to reach target sample size	___9 - 10_______

Methods: Assignment of interventions (for controlled trials)

Allocation:

Sequence generation	16a	Method of generating the allocation sequence (eg, computer-generated random numbers), and list of any factors for stratification. To reduce predictability of a random sequence, details of any planned restriction (eg, blocking) should be provided in a separate document that is unavailable to those who enrol participants or assign interventions	_____10________
Allocation concealment mechanism	16b	Mechanism of implementing the allocation sequence (eg, central telephone; sequentially numbered, opaque, sealed envelopes), describing any steps to conceal the sequence until interventions are assigned	_____10_______
Implementation	16c	Who will generate the allocation sequence, who will enrol participants, and who will assign participants to interventions	_____10________
Blinding (masking)	17a	Who will be blinded after assignment to interventions (eg, trial participants, care providers, outcome assessors, data analysts), and how	_____10________
	17b	If blinded, circumstances under which unblinding is permissible, and procedure for revealing a participant's allocated intervention during the trial	____NA_______

Methods: Data collection, management, and analysis

Data collection methods	18a	Plans for assessment and collection of outcome, baseline, and other trial data, including any related processes to promote data quality (eg, duplicate measurements, training of assessors) and a description of study instruments (eg, questionnaires, laboratory tests) along with their reliability and validity, if known. Reference to where data collection forms can be found, if not in the protocol	___16 - 19______
	18b	Plans to promote participant retention and complete follow-up, including list of any outcome data to be collected for participants who discontinue or deviate from intervention protocols	___10, 16 - 17___

Data management	19	Plans for data entry, coding, security, and storage, including any related processes to promote data quality (eg, double data entry; range checks for data values). Reference to where details of data management procedures can be found, if not in the protocol	__17 - 18_______
Statistical methods	20a	Statistical methods for analysing primary and secondary outcomes. Reference to where other details of the statistical analysis plan can be found, if not in the protocol	__18 - 19_______
	20b	Methods for any additional analyses (eg, subgroup and adjusted analyses)	__18 - 19_______
	20c	Definition of analysis population relating to protocol non-adherence (eg, as randomised analysis), and any statistical methods to handle missing data (eg, multiple imputation)	__18 - 19_______
**Methods: Monitoring**			
Data monitoring	21a	Composition of data monitoring committee (DMC); summary of its role and reporting structure; statement of whether it is independent from the sponsor and competing interests; and reference to where further details about its charter can be found, if not in the protocol. Alternatively, an explanation of why a DMC is not needed	___19__________
	21b	Description of any interim analyses and stopping guidelines, including who will have access to these interim results and make the final decision to terminate the trial	___19__________
Harms	22	Plans for collecting, assessing, reporting, and managing solicited and spontaneously reported adverse events and other unintended effects of trial interventions or trial conduct	__ 20 - 21_______
Auditing	23	Frequency and procedures for auditing trial conduct, if any, and whether the process will be independent from investigators and the sponsor	___NA________
**Ethics and dissemination**			
Research ethics approval	24	Plans for seeking research ethics committee/institutional review board (REC/IRB) approval	__24__________
Protocol amendments	25	Plans for communicating important protocol modifications (eg, changes to eligibility criteria, outcomes, analyses) to relevant parties (eg, investigators, REC/IRBs, trial participants, trial registries, journals, regulators)	__19___________

For peer review only - http://bmjopen.bmj.com/site/about/guidelines.xhtml

Consent or assent	26a	Who will obtain informed consent or assent from potential trial participants or authorised surrogates, and how (see Item 32)	____9___________
	26b	Additional consent provisions for collection and use of participant data and biological specimens in ancillary studies, if applicable	___NA__________
Confidentiality	27	How personal information about potential and enrolled participants will be collected, shared, and maintained in order to protect confidentiality before, during, and after the trial	___17 - 18______
Declaration of interests	28	Financial and other competing interests for principal investigators for the overall trial and each study site	__ 24__________
Access to data	29	Statement of who will have access to the final trial dataset, and disclosure of contractual agreements that limit such access for investigators	___24___________
Ancillary and post-trial care	30	Provisions, if any, for ancillary and post-trial care, and for compensation to those who suffer harm from trial participation	___NA__________
Dissemination policy	31a	Plans for investigators and sponsor to communicate trial results to participants, healthcare professionals, the public, and other relevant groups (eg, via publication, reporting in results databases, or other data sharing arrangements), including any publication restrictions	__17 - 18_______
	31b	Authorship eligibility guidelines and any intended use of professional writers	___NA__________
	31c	Plans, if any, for granting public access to the full protocol, participant-level dataset, and statistical code	_____**24**_________
**Appendices**			
Informed consent materials	32	Model consent form and other related documentation given to participants and authorised surrogates	______________
Biological specimens	33	Plans for collection, laboratory evaluation, and storage of biological specimens for genetic or molecular analysis in the current trial and for future use in ancillary studies, if applicable	______________

*It is strongly recommended that this checklist be read in conjunction with the SPIRIT 2013 Explanation & Elaboration for important clarification on the items. Amendments to the protocol should be tracked and dated. The SPIRIT checklist is copyrighted by the SPIRIT Group under the Creative Commons "Attribution-NonCommercial-NoDerivs 3.0 Unported" license.

BMJ Open

Peer-led family-centered Problem Management Plus for Immigrants (PMP-I) for mental health promotion among immigrants in the United States: protocol for a pilot, randomized controlled feasibility trial

Journal:	*BMJ Open*
Manuscript ID	bmjopen-2022-061353.R1
Article Type:	Protocol
Date Submitted by the Author:	21-Mar-2022

Complete List of Authors:	Poudel-Tandukar, Kalpana ; University of Massachusetts Amherst, Elaine Marieb College of Nursing<br>Jacelon, Cynthia; University of Massachusetts Amherst, Elaine Marieb College of Nursing<br>Martell, Christopher; University of Massachusetts Amherst, Department of Psychological and Brain Sciences, College of Natural Sciences<br>Poudel, Krishna; University of Massachusetts Amherst, Department of Health Promotion and Policy, School of Public Health and Health Sciences<br>Rai, Shan; Bhutanese Christian Society of Western Massachusetts<br>Ramdam, Razu; Bhutanese Christian Society of Western Massachusetts<br>Laws, Holly; University of Massachusetts Amherst, Department of Psychological and Brain Sciences, College of Natural Sciences<br>Meyer, Jerrold; University of Massachusetts Amherst, Department of Psychological and Brain Sciences, College of Natural Sciences<br>Bertone-Johnson, Elizabeth; University of Massachusetts Amherst, Department of Biostatistics and Epidemiology, School of Public Health and Health Sciences<br>Hollon, Steven; Vanderbilt University, Department of Psychology

**Primary Subject Heading**:	Mental health
Secondary Subject Heading:	Epidemiology
Keywords:	EPIDEMIOLOGY, MENTAL HEALTH, PSYCHIATRY, PREVENTIVE MEDICINE

SCHOLARONE™ Manuscripts

Peer-led family-centered Problem Management Plus for Immigrants (PMP-I) for mental health promotion among immigrants in the United States: protocol for a pilot, randomized controlled feasibility trial

Kalpana Poudel-Tandukar[1], Cynthia S Jacelon[1], Christopher R Martell[2], Krishna C Poudel[3], Shan Rai[4], Razu Ramdam[4], Holly Laws[2], Jerrold S Meyer[2], Elizabeth R. Bertone-Johnson[5], Steven D Hollon[6]

[1]Elaine Marieb College of Nursing, University of Massachusetts Amherst, MA, USA

[2]Department of Psychological and Brain Sciences, College of Natural Sciences, University of Massachusetts Amherst, MA, USA

[3] Department of Health Promotion and Policy, School of Public Health and Health Sciences, University of Massachusetts Amherst, MA, USA

[4]Bhutanese Christian Society of Western Massachusetts, Westfield, MA, USA

[5]Department of Biostatistics and Epidemiology, School of Public Health and Health Sciences, University of Massachusetts Amherst, MA, USA

[6]Department of Psychology, Vanderbilt University, Nashville, TN, USA

*Correspondence to: Kalpana Poudel-Tandukar, Ph.D., MPH, MPHC, CGM

Associate Professor, Elaine Marieb College of Nursing, University of Massachusetts Amherst, 220 Skinner Hall, 651 North Pleasant St, Amherst, MA 01003-9299, USA

Tel: 1-413-545-5095; Fax: 1-413-577-2500

Email: kalpana@umass.edu

For peer review only - http://bmjopen.bmj.com/site/about/guidelines.xhtml

Abstract

Introduction

Research is needed to investigate preventive strategies to reduce mental health burden and assess effective implementation among immigrants. Problem Management Plus (PMP) is a low-intensity multicomponent psychological intervention developed by the World Health Organization that trained laypeople can deliver. PMP has been adapted as a prevention intervention and developed as Problem Management Plus for Immigrants (PMP-I), including psychoeducation, problem-solving, behavioral activations, and mind-body exercise, to address immigrants' multiple stressors. This pilot trial aims to assess the feasibility and acceptability of PMP-I and provide a preliminary estimate of the difference between PMP-I vs. community support services pamphlets on the primary outcomes of interest (stress, anxiety, and depressive symptoms) to inform the design of a large-scale intervention.

Methods and analysis

The feasibility and acceptability of PMP-I will be assessed by measuring recruitment, session attendance, retention rates, program acceptability, and the fidelity of intervention delivery. This pilot trial will test preliminary effects of PMP-I vs. community support services pamphlets in a randomized controlled trial (*N= 232* participants from 116-families (2 members/family); 58 families randomized to condition intervention or control) on stress, anxiety, and depressive symptoms (primary outcomes), chronic physiological stress assessed in hair cortisol (secondary outcomes), and coping, family conflict resolution, and social networking (targets), with assessment at baseline, post-intervention, and 3-month post-intervention. Eligibility criteria for the primary study participants include Bhutanese ≥ 18 years resettled in Massachusetts with a score of ≤ 14 on

For peer review only - http://bmjopen.bmj.com/site/about/guidelines.xhtml

the Patient Health Questionnaire-9. All family members will be invited to participate in the family-based intervention (1-session/week for 5-week). Multilevel modeling will compare the longitudinal change in outcomes for each treatment arm.

Ethics and dissemination

The Institutional Review Board of the University of Massachusetts Amherst approved this study (Protocol: 1837). Written informed consent will be obtained from all participants. The study results will be used to inform the design of a large-scale intervention and will be disseminated in peer-reviewed journals and conferences.

Trial registration number: NCT04453709. Prospectively registered on 1st July 2020.

Keywords: Anxiety; Depression; Emotional Wellbeing; Mind-Body Intervention; Refugees; Social Wellbeing; Stress

Strengths and limitations of this study

- Whereas existing mental health interventions for immigrants are primarily based on treatment models to improve the access and quality of care for those with diagnosed mental health problems, this study is focused on developing, implementing, and pilot testing the effect of a culturally-tailored preventative behavioral intervention to reduce stress and prevent mental health problems among immigrants.
- This study includes culturally-tailored psychoeducation, behavioral activation, problem-solving, and mind-body interventions that could help to address multiple psycho-socio-cultural stressors through revitalizing resources at the individual, family, and community levels.

For peer review only - http://bmjopen.bmj.com/site/about/guidelines.xhtml

- The proposed intervention will be delivered to participants in their family environment by interventionists from the same community they trust and understand their language and problems from their cultural lens.
- This study will be among the first to link a preventive intervention with both biomarkers of stress (hair cortisol) and perceived stress and, using longitudinal data, to examine change over time in stress.
- Though clinical diagnosis is the gold standard, such an approach is not feasible in community-based studies, so this study relies on self-report measures of anxiety and depressive symptoms.

For peer review only

Introduction

Refugees resettled in the United States are vulnerable to mental health problems,[1, 2] such as anxiety and depression due to stress resulting from integrating into a new culture.[3-5] Refugees' risk for mental health problems increases during their acculturative process due to exposure to multiple stressors, such as adjustment to a new culture with limited language and socio-cultural skills, perceived discrimination, and a lack of culturally mediated and protective social support resources.[5, 6] Although mental health treatments are available to help alleviate the intrapersonal, social, and economic costs of mental disorders, refugees greatly underutilize these services.[1, 7, 8] Thus, evidence-based, culturally tailored preventative mental health interventions are needed for the growing number of refugees in the United States.

Existing interventions are focused explicitly on treatment models to provide quality care for those with diagnosed mental health problems[9] that do little to help reduce stress and prevent mental disorders for those who have not yet developed diagnosable symptoms. For prevention, a culturally tailored intervention that addresses multiple psycho-socio-cultural stressors, including social and cultural integration, holds good promise.[10, 11] Community-based preventative interventions that

For peer review only - http://bmjopen.bmj.com/site/about/guidelines.xhtml

promote positive impacts of social and cultural behaviors on mental health outcomes by protective resources are needed for the growing number of refugees dealing with life complexities.[12, 13] A review of community-based mental health interventions in refugees resettled to the United States suggests[14] that counseling, health promotion, and skill-building workshops facilitated by refugee peers[15-17] are helpful to reduce the psychological distress of many refugees who may be struggling with individual or family difficulties. Specifically, the Centers for Disease Control and Prevention (CDC) recommends using a non-clinical, community support approach to prevent mental illness among refugees resettled in the United States.[18]

Problem Management Plus (PMP) is a low-intensity evidence-based psychological intervention developed by the World Health Organization (WHO) that trained laypeople can deliver.[19, 20] PMP systematically teaches four strategies: stress management through mind-body exercises,[21-24] problem-solving,[25] behavioral activations,[26-33,] and skills to strengthen social support for individuals with psychological distress. PMP has been proven successful in reducing depression for women with mental disorders in Pakistan in a group setting.[34] We have adapted PMP to develop our Problem Management Plus for Immigrants (PMP-I) following a successful result of a pilot social and emotional wellbeing intervention. The pilot intervention included psychoeducation, mind-body exercise, problem-solving, and social support and reduced more than 50% prevalence of anxiety and depression from pre- to post-intervention among Bhutanese refugees when delivered in either a group[35] or a family setting.[36] While promising, these pilot results were drawn from only those receiving the treatment; no control group was available for comparison. Thus, the present study is to apply the adapted PMP in a randomized controlled trial. The present study is indicated for several reasons: our intervention model demands integration of social and emotional stressors; promising results of PMP in a non-controlled pilot study; the need to test the efficacy of PMP using

For peer review only - http://bmjopen.bmj.com/site/about/guidelines.xhtml

the more rigorous randomized controlled trial (RCT) study design; strong evidence of family and community ties in health care process; and growing consensus among community, scientists, and policymakers on the need for family-based care models that are sustainable.

Objectives and hypothesis

The main objectives of this study are:

a) To assess the feasibility and acceptability of PMP-I: i) recruitment, session attendance, retention rates, and program acceptability; ii) feasibility of measures for assessing inclusion/exclusion and fidelity of intervention delivery, and iii) barriers and facilitators of intervention using interview and focus group discussion with participants and facilitators.

b) To test the preliminary outcomes of PMP-I among Bhutanese adults 18 years or older living in Massachusetts with a score of 14 or below on the Patient Health Questionnaire-9 with trained community facilitators. Our central hypothesis is that PMP-I will reduce stress, anxiety, and depressive symptoms. We will test preliminary effects of PMP-I vs. community support services pamphlets in a randomized pilot trial (N=232 participants from 116 families (two members per family); 58 families per intervention and control) on perceived stress,[37] anxiety and depressive symptoms (primary outcomes).[38] chronic physiological stress assessed in hair cortisol (secondary outcome), and self-efficacy,[39] coping,[40] family conflict resolution,[41] family satisfaction,[42] social support (targets),[43] and social networks[44] with assessments at baseline, post-, and 3-month post-intervention.

Methods and analysis

Design and setting

This study will be conducted among resettled Bhutanese adults living in Massachusetts. Since 2008, Bhutanese people have been resettled in various states of the United States and are one of the largest groups of South Asian refugees (about 90 000).[45] They bear a high burden of mental health problems both in the nation (depression: 20%; suicide rate: 21.5 per 100 000)[18] and in western Massachusetts (depression: 23.8%; anxiety: 34.5%).[46] Given the importance of family relationships, communication, and coping in mental health,[47] the preventative social and emotional wellbeing intervention was designed for resettled Bhutanese adults in western Massachusetts using a community-based participatory research (CBPR) approach.[35]

This mixed-methods study will incorporate a two-arm randomized controlled feasibility trial and qualitative evaluation of PMP-I intervention's acceptability to a range of stakeholders. The study protocol has been reported following the Standard Protocol Items: Recommendations for Interventional Trials (SPIRIT). **Figure 1** shows the study flowchart, and **Table 1** shows an overview of study measures.

Participant recruitment

Participant inclusion criteria using a screening measure

This study will include eligible parents and adult children aged 18 and above interested in participating as primary study participants. At baseline, we will use a screening tool to identify individuals without significant depressive symptoms, as we aim to evaluate the effect of our intervention to prevent depression rather than treat depression. Eligibility criteria for our primary study participants include Bhutanese adults 18 years or older (both parents and children of each family) resettled in Massachusetts with a score of 14 or below on the Patient Health Questionnaire (PHQ-9), a screening questionnaire for depression. Our statistical analysis will focus on data from

For peer review only - http://bmjopen.bmj.com/site/about/guidelines.xhtml

For peer review only

primary study participants only with baseline PHQ-9 scores 14 or below. However, all other interested adult family members, both parents and their adult children, regardless of PHQ-9 score, will be invited to participate in the intervention. The PHQ-9 scores of participants will not be disclosed to anyone to maintain individual confidentiality. Besides, individuals with PHQ-9 screening scores '15-19' (moderately severe depression) and '20-27' (severe depression) will be provided with feedback on their screening questionnaire outcomes confidentially. They will be encouraged to consult their primary health care providers.

Participant exclusion criteria

Participants with clinically diagnosed mental disorders and those taking psychiatric medications for any mental health problems will also be encouraged to participate in the family-based intervention activities. However, in our primary statistical analysis, we will not consider data from those participants with PHQ-9 scores of 15 or above or diagnosed with mental health problems.

Informed consent

The principal investigator (PI) has prepared an informed consent document including an explanation on study background, screening, recruitment criteria, sample size, data collection, and intervention, study risks and benefits, confidentiality, National Institute of Mental Health Data Archive (NDA) data sharing policy, and hair samples collection procedure (online supplemental file). Trained community research assistants (RAs) will inform screening and study procedures to each participant using UMass Amherst Institutional Review Board (IRB)-approved single informed consent form visiting in-person. Once participants understand study details, RAs will request their signature or initials or fingerprint for those who cannot write in the consent form before data

For peer review only - http://bmjopen.bmj.com/site/about/guidelines.xhtml

collection. Participants will be reminded that their participation in the study is voluntary and free to leave the study without penalty.

Sample size and power calculations

The goal of the pilot project is to estimate the magnitude of the difference between the preventive intervention and the education control on the primary outcomes of interest to inform the design of a large-scale intervention. We conducted a power analysis to detect an effect size (ES) as small as ES=.30 with alpha = .05 and power of .80. We may find a larger effect in our pilot, but our understanding is that power estimates should be based on the smallest effect we want to detect rather than the size of the effect that we expect.[48] Analyses were performed using Optimal Design[49] by accounting for the intra-correlation among family members of .10 and alpha = .05, we would have 80% power to detect a standardized difference of ES=.30 between two treatment groups of 116 families (58 per treatment arm) with an equal probability of being randomized to each of our two intervention arms.

Randomization

We will randomly allocate selected families into intervention and control groups using a random sampling method after the baseline survey. RAs are unaware of which group the family will be randomized to when collecting baseline data. We will randomly assign 116 interested families (58 families per intervention and control) using a random number table. For random allocation, first, the PI will prepare the sampling frame that lists interested families, then assign a number to each family in the sampling frame, and finally select 116 numbers using a table of random numbers. We will

For peer review only - http://bmjopen.bmj.com/site/about/guidelines.xhtml

assign a random number selected at the first attempt for intervention and the second attempt for control.

Procedures are in place for tracking the participants for intervention and follow-up (e.g., contact address and phone). RAs will visit selected families and brief them about study procedures, informed consent, and procedures to protect human subjects. Two adult members of the selected families who meet the inclusion criteria and give informed consent will be recruited for the study. We will follow up with all families randomized to either study arm. We will not follow up with participants if they decide to end their participation at a particular time point of our study. But, we will include their already collected data in our analysis. Given our strong community networks and mobilization of community RAs, we anticipate low attrition rates in practice.

Problem Management Plus for Immigrants (PMP-I)

PMP-I is a 5-week, peer-led, culturally tailored mental health promotion program that includes psychoeducation, problem-solving, behavioral activation (90 minutes), breathing exercises, and yoga (90 minutes) in a family setting. PMP-I will use a structured approach, including once-a-week face-to-face sessions, yoga practice, breathing exercises, homework that includes practice activities, rebuilding individual skills, or learning new skills to reduce stress.

Our intervention aims to develop skills in coping adaptively in a new culture, seeking help and support for mental health problems, and other life skills opportunities that can improve their quality of life. **Module 1**: **Managing Stress** includes yoga, breathing exercise, stress-management sessions, and practice exercises to develop coping strategies that are most helpful to reduce stress and then plan a strategy to carry out those solutions. **Module 2: Managing Problems** includes practice exercises to identify the problems causing the most concern, develop solutions that are

For peer review only - http://bmjopen.bmj.com/site/about/guidelines.xhtml

most helpful in addressing the problem, and then plan a strategy to carry out those solutions. **Module 3: Behavioral Activation** includes communication skill sessions and practice exercises to identify pleasant activities (time to yourself; connecting with others; self-care), breaking down the task into smaller steps, and schedule tasks, and then plan a strategy to carry out those tasks. **Module 4: Strengthening Social Support** includes social skills sessions and practice exercises to identify at least one person or service from whom the participant feels comfortable getting some support, and to plan exactly what the participant is going to do, and then schedule a day to carry out the tasks. **Module 5: Staying Well** includes practice exercises to make a plan that helps to create a supportive family environment.

Community interventionists (CIs) are trained community members with at least a high school level of education, and no formal training or prior experience with mental health will deliver the PMP-I. Dr. Christopher Martell, board-certified in behavioral and cognitive psychology and clinical psychology and a Massachusetts Licensed Psychologist, will provide 12 days of training to the interventionists in collaboration with the PI and Dr. Steven D. Hollon (Professor of Psychiatry, Psychology and Human Development) following the World Health Organization PMP Helpers' Training Guide[50] adapted for PMP-I. Classroom training includes information about stress, depression, mental health problems, the rationale for each intervention strategy, necessary helping skills, practice plan formulation, role-plays, peer observations, and group discussion related to core intervention concepts, practices, and supervision skills. Supervision involves discussing participants' progress and difficulties experienced when delivering strategies and role-playing on managing problems or practicing skills. They will use the PMP-I intervention manual to provide PMP-I to community members in family settings under field supervisors and PI's supervision. We will conduct a formal evaluation of the interventionists' readiness to implement/supervise the PMP-I

For peer review only - http://bmjopen.bmj.com/site/about/guidelines.xhtml

intervention, such as using the manual, answering questions, managing time, using a fidelity checklist, practicing exercise, and role play to provide feedback as necessary.

A licensed yoga trainer will provide 4 hours of breathing exercises and 16 hours of yoga to CIs and field supervisors using a mind-body exercise training manual. Classroom training includes theoretical and practices to guide participants in mind-body exercises for attention to breath, body sensation, emotional awareness, and mental function on different postures of yoga practices such as *Pranayama* (3 poses) and *Asana* (21 poses). Training will include practice assessment at the end to ensure that all field staff is trained, using a checklist, practice exercise, and role-plays.

Community Support Service Program

Bhutanese community members expressed that knowing the health and life skill development program available in their communities would benefit them in strengthening their life skills.[51] By considering their request, we have prepared pamphlets including names, contact, and service details of community and health organizations in the area where they live. CIs will distribute community support service program pamphlets to control families.

Feasibility and acceptability assessment

The PMP trainer's training guidelines provide specific tools for evaluating and monitoring the intervention, which we use to monitor intervention delivery fidelity. These tools are PMP Quiz, PMP Helper's Supervision Form, PMP Helper Classroom-based Competency Assessment, PMP Helper In-field based Competency Assessment, PMP Trainer/Supervisor Competency Assessment, and Session-by-Session Checklists for PMP Helpers.[50] We have adapted these tools in the context of our program contents. Using these standard tools, we will evaluate session-by-session classroom

For peer review only - http://bmjopen.bmj.com/site/about/guidelines.xhtml

and in-field based competencies of community interventionists and field supervisors and provide them feedback as needed using supervision forms, role-plays, group discussion, and training.

At the field level, field supervisors will monitor intervention sessions delivered by community interventionists using standard checklists. Items include adherence to the manual, percent of intervention content administered, proper use of time/materials, and adequate response to participants' questions. They will also monitor participants' engagement, acceptability, and satisfaction via brief questionnaires with participants and interventionists during and after intervention completion. Moreover, community interventionists will be asked to complete a structured checklist on the attendance, compliance, and satisfaction towards intervention components immediately after each session.

The PI will conduct a focus group discussion (FGD) in the Nepali language with interventionists, supervisors, and participants separately to collect information on barriers and facilitators of intervention, perceptions about whether the intervention met participants' needs, and feedback on how effectively the program team worked with participants. Interviews and FGD will be documented verbatim in a written transcript for subsequent analysis. All qualitative data will be analyzed using thematic content analysis.[52] Feedback provided by the field staff will be reviewed and coded to identify recurrent themes regarding the intervention's acceptability. Fidelity data will be used to assess intervention content and transmission.

Primary outcome measures

Anxiety and Depressive Symptoms

The Hopkins Symptom Checklist-25 (HSCL-25) will be used to measure anxiety, and depressive symptoms experienced over the past month.[38] It is composed of a 10-item subscale for anxiety and

For peer review only - http://bmjopen.bmj.com/site/about/guidelines.xhtml

a 15-item subscale for depression, with each item scored on a Likert scale from 1 (not at all) to 4 (extremely). The scale has high internal consistency (Cronbach's α) for anxiety (0.95) and depression (0.94) in the Bhutanese study.[35]

Perceived Stress

The 10-item Cohen Perceived Stress Scale (PSS) will be used to assess perceived stress.[37] The PSS uses a 5-point Likert scale (ranging from 0, "never" to 4, "very often") to assess psychological stress experienced during the past month, including the extent to which situations felt unpredictable, uncomfortable, and overwhelming. In the Bhutanese study, the scale has high internal consistency (Cronbach's $\alpha = 0.80$).[35]

Secondary outcome measures

Physiological stress

We will use the enzyme-linked immunosorbent assay (ELISA) cortisol hair test (average hormone levels over the past three months) as a biomarker to measure physiological stress. Hair samples will be processed in the neuroendocrine lab at the University of Massachusetts Amherst.[53, 54] Sensitive and specific enzyme immunoassay (Arbor Assays) will be used for the analysis. The assay has intra- and inter-assay coefficients of variation of <10%.

Other measures

Coping strategy

Coping strategy will be measured using a 32-item Coping Strategies Inventory-Short Form (CSI-SF).[40] The CSI-SF includes two overall coping factors, Engagement and Disengagement, and four

For peer review only - http://bmjopen.bmj.com/site/about/guidelines.xhtml

secondary factors, Problem Engagement, Problem Disengagement, Emotion Engagement, and Emotion Disengagement. The CSI-SF scale (Cronbach's α = 0.95) has high internal consistencies in the Bhutanese study.[35] Participants were asked to rate their responses on a 5-point Likert-type scale ranging from *not at all (1)* to *very much (5)*.

Coping Self-efficacy

Self-efficacy will be measured using a 26-item Coping Self-efficacy (CSE) scale for coping with challenges and threats.[39] Each item of the scale will be rated on an 11-point scale Likert-type scale ranging from *(0) cannot do at all, (5) moderately certain can do, and (10) certain can do*. The scale has high internal consistency (Cronbach's α = 0.96) in the previous Bhutanese study.[35]

Social support

Perceived social support will be measured using a 12-item Multidimensional Scale of Perceived Social Support (MSPSS),[43] including support from friends, family, and significant others. A sample item for this scale is, "My family tries to help me." Each item of the scale will be rated on a 5-point Likert-type scale ranging from *strongly disagree (1)* to *strongly agree (5)*. Graded items will be summed up to provide a total score, and higher scores indicate high social support. The scale has high internal consistency (Cronbach's α = 0.92) in the previous Bhutanese study.[35]

Social network

We will use a Lubben Social Network Scale-Revised (LSNS-R) to measure social networks among family and friendships.[44] It consists of six questions, which assess kinship ties, and a comparable set of six questions, which determine friend ties by replacing the word relatives with the word friends.

For peer review only - http://bmjopen.bmj.com/site/about/guidelines.xhtml

We prepared three questions to measure cross-cultural social ties following a similar pattern. The scale has high internal consistency for kinship ties (Cronbach's $\alpha = 0.78$), friendship ties (Cronbach's $\alpha = 0.80$), and cross-cultural social ties (Cronbach's $\alpha = 0.74$) in the previous Bhutanese study.[35] These items will be scored on a five-point Likert scale ranging from *none (0)* to *9 or more or always (5)*.

Family conflict resolution

Family conflict resolution, including positive or negative resolution, effective communication, and discussion of differences, will be measured using a 17-item version of the "Family Conflict Resolution" scale.[41] This scale has high internal consistency (Cronbach's $\alpha = 0.90$) in the previous Bhutanese study.[35] Participants will be asked to respond on a 7-point Likert-type scale, ranging from *never (1)* to *always (7)*.

Family satisfaction: Family satisfaction with various aspects of family functioning, including family closeness, flexibility, and communication, will be measured using a 10-item family satisfaction scale.[55] Participants will be asked to respond on a 5-point Likert-type scale, ranging from *very dissatisfied (1)* to *extremely satisfied (5)*.

Data management

All interviews will be conducted with the utmost privacy and confidentiality. Each interested and eligible adult participant in the family will be interviewed individually, in a private place where they feel comfortable, by our trained community RAs. The RAs will ensure audio and visual privacy at these sites, and ensure data confidentiality. RAs will reassure participants that numerical

For peer review only - http://bmjopen.bmj.com/site/about/guidelines.xhtml

For peer review only

codes would be used in place of names in all records to ensure confidentiality. The survey materials (questionnaires, transcriptions, and field notes) will be stored in a locked cabinet in the PI's office. Data entry will be done on the PI's office computer (encrypted and password protected) under the full supervision of the PI. The original data will be kept on OneDrive, a secure, networked university data storage system. De-identified data sets will be used for statistical analyses. The PI herself will do data analysis and documentation. All information will be presented in aggregate form in the manuscript or conference abstract, and no individual respondent will be identified.

Data analysis

We will compare baseline characteristics of intervention and control groups using chi-square and t-tests as appropriate. While differences between groups are not expected because of the randomization used in the study design, variables showing significant differences between the two groups will be included as covariates in primary analyses. The primary analyses will test whether participants' outcomes in the PMP-I arm differ from those in the control arm. Multilevel modeling will compare outcomes of each treatment arm while accounting for the clustering of participants within families. Continuous outcomes will be analyzed using hierarchical linear modeling, and dichotomous outcomes will be analyzed using multilevel generalized linear models with a Bernoulli distribution appropriate to nonlinear binary outcomes.[56]

We expect approximately 2 to 4 members for each of the 58 families in each treatment arm, and the correlation among family members' responses will be accounted for in the model. Hierarchical or multilevel modeling is suited to these data as it accounts for the clustering of members within families and unbalanced designs (i.e., different family sizes).[56] This will be an intention-to-treat type of analysis, as multilevel modeling allows retention of all participants irrespective of the

For peer review only - http://bmjopen.bmj.com/site/about/guidelines.xhtml

number of sessions attended (multilevel modeling uses maximum likelihood estimation, one of the recommended ways of handling missing data). The analysis will estimate endpoint outcomes based on repeated measures (Level 1) within individuals (Level 2) within families (Level 3). Separate models will be created to evaluate the relationships between mediators (targets) and outcomes and explore mediators (e.g., coping) of intervention-outcome relation. All analyses will be performed using SAS, version 9 (SAS Institute Inc, Cary, NC).

Independent Safety Monitor

We will select an Independent Safety Monitor (ISM) with mental health expertise, whose primary responsibility is to provide independent monitoring of this clinical trial in a timely fashion. Overall, the ISM will review enrollment data, safety data, and data integrity to maintain safety in the trial. The PI will submit data reports once a year to the ISM. The report will include the key variables necessary for monitoring the safety and quality of data collection and the integrity of the study, including inclusion criteria, informed consent, subject enrollment and retention, data confidentiality, intervention compliance, dropouts, adverse events, protocol compliance, data quality, and baseline characteristics of study participants. The ISM will have access to all safety and data quality information collected and will have the authority to stop the study if it is determined that there are unacceptable risks to participants. The ISM also will review the study protocol, informed consent, and all relevant documents before the onset of the study and will review and approve amendments to these documents. The ISM will issue a monitoring report to the PI following each review. The PI will submit all review reports to the UMass Amherst IRB and NIMH Program Officer in annual progress reports.

Trial management

For peer review only - http://bmjopen.bmj.com/site/about/guidelines.xhtml

The PI will assume overall responsibility of trial management, working together with the entire research team throughout the project, meeting monthly with Co-Investigators (psychiatrist, cognitive behavior therapist, and epidemiologist), and once every week with field staff (supervisors, interventionists, and research assistants) via in-person or zoom or text message as needed. During the trial, experienced field supervisors from the Bhutanese community, who are trained as a community health workers and have worked with the PI in previous family-based mental health intervention studies with depressive and suicidal ideation outcomes, will take responsibility for the day-to-day oversight of the participants and field teams in the implementation of the trial. Field supervisors will immediately report any noted adverse events among participants to the PI. The PI will report adverse events data to the Independent Safety Monitor (ISM), UMass Amherst IRB, and National Institute of Mental Health (NIMH) Program Officer following NIMH guidelines for reportable events, as described below.

Adverse events reporting

Throughout the study period, all study participants will be monitored daily by the field supervisors under the PI's supervision. Field supervisors will request study participants and their family members to immediately report any unanticipated serious adverse events in their family, such as 1) reporting suicidal ideation or attempts, hospitalization, disability, and/or death; 2) discomfort with the PMP-I program content and/or evaluation procedures, and 3) risk of a breach of confidentiality, of the collected data and/or by program personnel to field supervisor or PI directly. Field supervisors will immediately report details of such adverse events to PI. The PI will be responsible for reporting them to the UMass Amherst IRB, ISM, and NIMH Program Officer by secure email within ten business days of the study team becoming aware of any serious adverse events. The PI

For peer review only - http://bmjopen.bmj.com/site/about/guidelines.xhtml

will be responsible for summarizing all adverse events that are deemed expected and/or unrelated to the study in the annual progress report submitted to the UMass Amherst IRB, ISM, and NIMH Program Officer by secure email.

Patient and public involvement

Patients or the public were not involved in the design, or conduct, or reporting, or dissemination plans of our research.

Ethics and dissemination

The Institutional Review Board of the University of Massachusetts Amherst approved this study (Protocol ID: 1837) and certified that it will be performed according to the ethical standards laid down in the 1964 Declaration of Helsinki and its later amendments.

Before enrolling participants in the study, written informed consent will be taken from each person after a complete description of the study. All participants will have the opportunity to discuss any questions or issues (online supplemental file).

The study data will be shared via the National Institute of Mental Health Data Archive. Access to data used in the proposed project will be considered for sharing in compliance with the NIH Grants Policy on Sharing Unique Research Resources. The study results will be used to inform the design of a large-scale intervention and will be disseminated in peer-reviewed journals and conferences.

Discussion

This study reports an RCT protocol that tests PMP-I's feasibility, acceptability, and preliminary outcomes with trained community facilitators. This study is built on prior research that has shown

For peer review only - http://bmjopen.bmj.com/site/about/guidelines.xhtml

the effectiveness of social and emotional wellbeing intervention, including psychoeducation, problem-solving, social support, and mind-body exercises, to reduce stress, anxiety, and depressive symptoms among Bhutanese adults resettled in MA at a group[35] and family settings[36] using a pre- and post-test intervention design. Our project designed for Bhutanese immigrants includes evidence-based interventions of specific relevance to this community, such as psychoeducation,[57] problem-solving,[25] behavioral activations,[26-33] mind-body exercises,[21-24] and strengthening social support to address identified social (e.g., social isolation, language difficulties) and emotional (e.g., lack of self-esteem or self-efficacy)[51] stressors by strengthening protective factors (e.g., resilience or coping).[58, 59] This study is innovative as it will be the first culturally tailored, preventive, family-based, multi-component behavioral intervention driven by the community to reduce stress. We will have pilot-tested a preventative mental health intervention for Bhutanese adults upon completion. This study can be expected to impact reducing stress and promoting immigrants' mental wellbeing.

Our project is likely to be replicated with other immigrant communities with minimal adaptation over the long term for three reasons. First, our intervention is guided by a strengths-based approach in which we plan to include community strengths. This principle can be applied to capture and integrate the strengths of any community. Second, our program prioritizes the training of community members as interventionists, as these are individuals whom the community trusts, who share the same cultural lens as the community and thus can well understand language and specific challenges, and who have a vested interest in the strength and resilience of their community. This aspect of our intervention design is easily adaptable to other populations. Finally, intervention is designed to be delivered in family settings where participants are most comfortable and family members can support each other throughout their lives. This component is crucial in collectivistic societies where family bonds and group identity are strong. Thus, our family-based strategies could

For peer review only - http://bmjopen.bmj.com/site/about/guidelines.xhtml

be replicable in other immigrant groups, where there are similarities in social and emotional stressors, challenges, community strengths (coping, resilience, social support), strong family support, and cultural preference of native community counselors for their mental health consultation. Our strength-based and peer-led strategies promote community engagement and make the intervention sustainable.[60-62]

Although our study design has several strengths, it also has some methodological limitations. We measured anxiety and depressive symptoms using the HSCL-25 scale, which was validated with clinical DSM-IV diagnoses of major depressive disorder[63] among refugees in Nepal[64] and other countries. [65] Although clinical diagnosis is the gold standard; such an approach is not feasible in community-based studies. We have started implementing an intervention during the COVID-19 pandemic, so we may need to be flexible in the time frame for conducting surveys and implementing intervention sessions while waiting for participants to recover from COVID-19 infections they might have contracted during the study period.

In conclusion, our trial will provide information on the feasibility of PMP-I among the immigrant population and effect size estimate to design a larger-scale randomized controlled trial intervention study.

Trial status

Recruitment of participants was delayed due to the COVID-19 pandemic and started on August 17, 2021. At the time of manuscript submission, trial was ongoing. Results of this study are expected in mid-2024.

List of abbreviations

For peer review only - http://bmjopen.bmj.com/site/about/guidelines.xhtml

CBPR: Community-Based Participatory Research; CDC: Centers for Disease Control and Prevention; CIs: Community Interventionists; CSE: Coping Self-efficacy; CSI-SF: Coping Strategies Inventory-Short Form; ELISA: Enzyme-Linked Immunosorbent Assay; ES: Effect Size; FGD: Focus Group Discussion; HSCL-25: Hopkins Symptom Checklist-25; IRB: Institutional Review Board; ISM: Independent Safety Monitor; LSNS-R: Lubben Social Network Scale-Revised; MSPSS: Multidimensional Scale of Perceived Social Support; NDA: National Institute of Mental Health Data Archive; NIMH: National Institute of Mental Health; PHQ-9: Patient Health Questionnaire; PI: Principal Investigator; PMP: Problem Management Plus; PMP-I: Problem Management Plus for Immigrants; PSS: Perceived Stress Scale; RAs: Research Assistants; RCT: Randomized Controlled Trial; SPIRIT: Standard Protocol Items: Recommendations for Interventional Trials; WHO: World Health Organization

Contributors

KPT conceived the study and drafted the study and trial protocols. All authors were involved in the design of the study; KPT, CSJ, CM, KCP, SR, RR, HL, JSM, ERBJ, and SDH were involved in revising the study protocol for ethics review, and all authors were involved in commenting on and revising the trial protocol. All authors read and approved the final manuscript.

Competing interests

The author(s) declared no potential conflicts of interest with respect to the research, authorship, and/or publication of this article.

Funding

For peer review only - http://bmjopen.bmj.com/site/about/guidelines.xhtml

This trial is funded by the National Institute of Mental Health of the National Institutes of Health under Award Number R34MH118396 (PI: Dr. Kalpana Poudel-Tandukar). The content is solely the authors' responsibility and does not necessarily represent the official views of the National Institutes of Health.

Consent for publication

Not applicable.

FIGURE TITLES

Figure 1. Study flowchart

References

1. Taylor EM, Yanni EA, Pezzi C, Guterbock M, Rothney E, Harton E, Montour J, Elias C, Burke H. Physical and mental health status of Iraqi refugees resettled in the United States. J Immigr Minor Health 2014;16:1130-7.

2. Wagner J, Burke G, Kuoch T, Scully M, Armeli S, Rajan TV. Trauma, healthcare access, and health outcomes among Southeast Asian refugees in Connecticut. J Immigr Minor Health 2013;15:1065-72.

3. Alemi Q, Stempel C. Discrimination and distress among Afghan refugees in northern California: The moderating role of pre- and post-migration factors. PLoS One 2018;13:e0196822.

4. Ellis BH, Lankau EW, Ao T, Benson MA, Miller AB, Shetty S, Lopes Cardozo B, Geltman PL, Cochran J. Understanding Bhutanese refugee suicide through the interpersonal-psychological theory of suicidal behavior. Am J Orthopsychiatry 2015;85:43-55.

For peer review only - http://bmjopen.bmj.com/site/about/guidelines.xhtml

For peer review only

5. Vonnahme LA, Lankau EW, Ao T, Shetty S, Cardozo BL. Factors Associated with Symptoms of Depression Among Bhutanese Refugees in the United States. J Immigr Minor Health 2015;17:1705-14.

6. Williams ME, Thompson SC. The use of community-based interventions in reducing morbidity from the psychological impact of conflict-related trauma among refugee populations: a systematic review of the literature. J Immigr Minor Health 2011;13:780-94.

7. Ellis BH, Lincoln AK, Charney ME, Ford-Paz R, Benson M, Strunin L. Mental health service utilization of Somali adolescents: religion, community, and school as gateways to healing. Transcult Psychiatry 2010;47:789-811.

8. Colucci E, Szware J, Minas H, Paxton G, Guerra C. The utilization of mental health services by children and young people from a refugee background: A systematic literature review. International Journal of Culture and Mental Health 2012;7:86-108.

9. Swinson Evans T, Berkman N, Brown C, Gaynes B, Palmieri Weber R. Disparities within serious mental illness. Technical Brief No. 25. (Prepared by the RTI International–University of North Carolina Evidence-based Practice Center under Contract No. 290-2015-00011-I). AHRQ Publication No.16-EHC027-EF. Rockville, MD: Agency for Healthcare Research and Quality; May 2016. , 2016.

10. Goodkind JR, Hess JM, Isakson B, LaNoue M, Githinji A, Roche N, Vadnais K, Parker DP. Reducing refugee mental health disparities: a community-based intervention to address postmigration stressors with African adults. Psychol Serv 2014;11:333-46.

11. Weine SM. Developing preventive mental health interventions for refugee families in resettlement. Fam Process 2011;50:410-30.

For peer review only - http://bmjopen.bmj.com/site/about/guidelines.xhtml

12. Kirmayer LJ, Narasiah L, Munoz M, Rashid M, Ryder AG, Guzder J, Hassan G, Rousseau C, Pottie K. Common mental health problems in immigrants and refugees: general approach in primary care. CMAJ 2011;183:E959-67.

13. Pumariega AJ, Rothe E, Pumariega JB. Mental health of immigrants and refugees. Community Ment Health J 2005;41:581-97.

14. Peterson C, Poudel-Tandukar K, Sanger K, Jacelon CS. Improving Mental Health in Refugee Populations: A Review of Intervention Studies Conducted in the United States. Issues Ment Health Nurs 2020;41:271-82.

15. Im H, Rosenberg R. Building Social Capital Through a Peer-Led Community Health Workshop: A Pilot with the Bhutanese Refugee Community. J Community Health 2016;41:509-17.

16. Birman D, Beehler S, Harris EM, Everson ML, Batia K, Liautaud J, Frazier S, Atkins M, Blanton S, Buwalda J, Fogg L, Cappella E. International Family, Adult, and Child Enhancement Services (FACES): a community-based comprehensive services model for refugee children in resettlement. Am J Orthopsychiatry 2008;78:121-32.

17. Boehnlein JK, Kinzie JD, Sekiya U, Riley C, Pou K, Rosborough B. A ten-year treatment outcome study of traumatized Cambodian refugees. J Nerv Ment Dis 2004;192:658-63.

18. CDC. Suicide and suicidal ideation among Bhutanese refugees--United States, 2009-2012. MMWR Morb Mortal Wkly Rep 2013;62:533-6.

19. Dawson KS, Bryant RA, Harper M, Kuowei Tay A, Rahman A, Schafer A, van Ommeren M. Problem Management Plus (PM+): a WHO transdiagnostic psychological intervention for common mental health problems. World Psychiatry 2015;14:354-7.

For peer review only

For peer review only - http://bmjopen.bmj.com/site/about/guidelines.xhtml

20. World Health Organization. Problem Management Plus (PM+): Individual psychological help for adults impaired by distress in communities exposed to adversity. (Generic field-trial version 1.0). Geneva, WHO, 2016.

21. Wahbeh H, Elsas SM, Oken BS. Mind-body interventions: applications in neurology. Neurology 2008;70:2321-8.

22. Falsafi N. A Randomized Controlled Trial of Mindfulness Versus Yoga: Effects on Depression and/or Anxiety in College Students. J Am Psychiatr Nurses Assoc 2016;22:483-97.

23. Prathikanti S, Rivera R, Cochran A, Tungol JG, Fayazmanesh N, Weinmann E. Treating major depression with yoga: A prospective, randomized, controlled pilot trial. PLoS One 2017;12:e0173869.

24. de Manincor M, Bensoussan A, Smith C, Fahey P, Bourchier S. Establishing key components of yoga interventions for reducing depression and anxiety, and improving well-being: a Delphi method study. BMC Complement Altern Med 2015;15:85.

25. Cuijpers P, de Wit L, Kleiboer A, Karyotaki E, Ebert DD. Problem-solving therapy for adult depression: An updated meta-analysis. Eur Psychiatry 2018;48:27-37.

26. Martell CR, Dimidjian S, Hermann-Dunn R. Behavioral activation for depression: a clinician's guide. Gullford New York, NY.; 2010.

27. Mazzucchelli T, R. K, Rees C. Behavioral activation treatments for depression in adults: a meta-analysis and review. . Clin Psychol Sci Pract 2009;16:383-411.

28. Kanter JW, Manos RC, Bowe WM, Baruch DE, Busch AM, Rusch LC. What is behavioral activation? A review of the empirical literature. Clin Psychol Rev 2010;30:608-20.

29. Cuijpers P, van Straten A, Warmerdam L. Behavioral activation treatments of depression: a meta-analysis. Clin Psychol Rev 2007;27:318-26.

For peer review only - http://bmjopen.bmj.com/site/about/guidelines.xhtml

30. Cuijpers P, van Straten A, Warmerdam L. Problem solving therapies for depression: a meta-analysis. Eur Psychiatry 2007;22:9-15.

31. Kanter JW, Bush AM, Rusch LC. Behavioural activation: the CBT distinctive feature series. New York: Routledge.; 2009.

32. Veale D. Behavioural activation for depression. Adv Psychiatr Treat 2008;14:29-36.

33. Chowdhary N, Anand A, Dimidjian S, Shinde S, Weobong B, Balaji M, Hollon SD, Rahman A, Wilson GT, Verdeli H, Araya R, King M, Jordans MJ, Fairburn C, Kirkwood B, Patel V. The Healthy Activity Program lay counsellor delivered treatment for severe depression in India: systematic development and randomised evaluation. Br J Psychiatry 2015;208:381-8.

34. Khan MN, Hamdani SU, Chiumento A, Dawson K, Bryant RA, Sijbrandij M, Nazir H, Akhtar P, Masood A, Wang D, Wang E, Uddin I, Ommeren MV, Rahman A. Evaluating feasibility and acceptability of a group WHO trans-diagnostic intervention for women with common mental disorders in rural Pakistan: a cluster randomised controlled feasibility trial. Epidemiol Psychiatr Sci 2017:1-11.

35. Poudel-Tandukar K, Jacelon CS, Rai S, Ramdam P, Bertone-Johnson ER, Hollon SD. Social and Emotional Wellbeing (SEW) Intervention for Mental Health Promotion Among Resettled Bhutanese Adults in Massachusetts. Community Ment Health J 2021;57:1318-27.

36. Poudel-Tandukar K, Jacelon CS, Poudel KC, Bertone-Johnson ER, Rai S, Ramdam P, Hollon SD. Mental health promotion among resettled Bhutanese adults in Massachusetts: Results of a peer-led family-centred Social and Emotional Well-being (SEW) intervention study. Health Soc Care Community 2021.

37. Cohen S, Kamarck T, Mermelstein R. A global measure of perceived stress. J Health Soc Behav 1983;24:385-96.

For peer review only

For peer review only - http://bmjopen.bmj.com/site/about/guidelines.xhtml

38. Derogatis LR, Lipman RS, Rickels K, Uhlenhuth EH, Covi L. The Hopkins Symptom Checklist (HSCL): a self-report symptom inventory. Behav Sci 1974;19:1-15.

39. Chesney MA, Neilands TB, Chambers DB, Taylor JM, Folkman S. A validity and reliability study of the coping self-efficacy scale. Br J Health Psychol 2006;11:421-37.

40. Tobin DL. Coping strategies therapy for bulimia nervosa. Washington, DC: American Psychological Association Press, 2000., 2000.

41. Roskos PT, Handal PJ, Ubinger ME. Family Conflict Resolution: Its Measurement and Relationship with Family Conflict and Psychological Adjustment. Psychology 2010;1:370-76.

42. Olson DH. Family Satisfaction Scale. Minneapolis, MN: Life Innovations, Inc.; 2010.

43. Zimet GD, Dahlem NW, Zimet SG, Farley GK. The multidimensional scale of perceived social support. Journal of Personality Assessment 1988;52:30-41.

44. Lubben J, Blozik E, Gillmann G, Iliffe S, von Renteln Kruse W, Beck JC, Stuck AE. Performance of an abbreviated version of the Lubben Social Network Scale among three European community-dwelling older adult populations. Gerontologist 2006;46:503-13.

45. United States Embassy Kathmandu. U.S. Ambassador Bids Farewell to 90,000th Refugee to Resettle to the United States. 21 September, 2016. Accessed July 24, 2017. From: https://np.usembassy.gov/u-s-ambassador-bids-farewell-90000th-refugee-resettle-united-states/ 2016.

46. Poudel-Tandukar K, Bertone-Johnson ER, KC P, Poudel KC. Family-engaged preventative behavioral interventions are needed to promote mental health among HIV-infected individuals living in resource-poor settings. American Public Health Association Annual Meeting and Exposition (APHA), Philadelphia, USA. November 2-6, 2019. (Abstract Number: 438575): Oral Presentation. 2019.

For peer review only

For peer review only - http://bmjopen.bmj.com/site/about/guidelines.xhtml

47. Slobodin O, de Jong JT. Family interventions in traumatized immigrants and refugees: A systematic review. Transcult Psychiatry 2015;52:723-42.

48. Kraemer HC, Mintz J, Noda A, Tinklenberg J, Yesavage JA. Caution regarding the use of pilot studies to guide power calculations for study proposals. Arch Gen Psychiatry 2006;63:484-9.

49. Raudenbush, S. W., et al. (2011). Optimal Design Software for Multi-level and Longitudinal Research (Version 3.01) [Software]. Available from www.wtgrantfoundation.org.

50. World Health Organization. Problem Management Plus (PM+) Training Guide for PM+ Helpers: To accompany the PM+ intervention manual: Problem Management Plus: Individual psychological help for adults impaired by distress in communities exposed to adversity –Field Version 1.0 WHO (2016). Series on Low-Intensity Psychological Interventions – 2. Geneva, WHO, 2018. .

51. Poudel-Tandukar K, Jacelon CS, Chandler GE, Gautam B, Palmer PH. Sociocultural Perceptions and Enablers to Seeking Mental Health Support Among Bhutanese Refugees in Western Massachusetts. Int Q Community Health Educ 2019;39:135-45.

52. Green J, Thorogood N. Qualitative methods for health research. Thousands Oaks, CA, USA: SAGE Publishing; 2014. 2014.

53. Meyer J, Novak M, Hamel A, Rosenberg K. Extraction and analysis of cortisol from human and monkey hair. J Vis Exp 2014:e50882.

54. Meyer JS, Novak MA. Minireview: Hair cortisol: a novel biomarker of hypothalamic-pituitary-adrenocortical activity. Endocrinology 2012;153:4120-7.

55. Olson DH, Wilson M. Family Satisfaction. In Olson, D.H., and colleagues. Families: What Makes Them Work. Newbury Park, CA: Sage Publishing.; 1982, 1989.

For peer review only - http://bmjopen.bmj.com/site/about/guidelines.xhtml

56. Raudenbush S, W., Bryk. AS. Hierarchical Linear Models: Applications and Data Analysis Methods. 2nd ed. Thousand Oaks: Sage Publications. 2002.

57. Zlotnick C, Johnson SL, Miller IW, Pearlstein T, Howard M. Postpartum depression in women receiving public assistance: pilot study of an interpersonal-therapy-oriented group intervention. Am J Psychiatry 2001;158:638-40.

58. Poudel-Tandukar K, Jacelon CS, Bertone-Johnson ER, Gautam B, Palmer PH, Hollon SD. Coping strategies and stress among resettled Bhutanese adults in Massachusetts. Am J Orthopsychiatry 2020;90:502-09.

59. Poudel-Tandukar K, Chandler GE, Jacelon CS, Gautam B, Bertone-Johnson ER, Hollon SD. Resilience and anxiety or depression among resettled Bhutanese adults in the United States. Int J Soc Psychiatry 2019:20764019862312.

60. Singla DR, Kohrt BA, Murray LK, Anand A, Chorpita BF, Patel V. Psychological Treatments for the World: Lessons from Low- and Middle-Income Countries. Annu Rev Clin Psychol 2017;13:149-81.

61. Webel AR, Okonsky J, Trompeta J, Holzemer WL. A systematic review of the effectiveness of peer-based interventions on health-related behaviors in adults. Am J Public Health 2010;100:247-53.

62. Petosa R, Smith LH. Peer mentoring for health behaviors change: A systematic review. . Am J Health Educ 2014;45:351-57.

63. APA. *Diagnostic and statistical manual of mental disorder*, 4th ed. DSM-IV. 1994 APA. https://doi.org/10.1146/annur ev-clinpsy-032816-045217

64. Shrestha NM, Sharma B, Van Ommeren M, Regmi S, Makaju R, Komproe I, Shrestha GB, de Jong JTVM. Impact of torture on refugees displaced within the developing world:

For peer review only - http://bmjopen.bmj.com/site/about/guidelines.xhtml

Symptomatology among Bhutanese refugees in Nepal. JAMA 1998;280:443–448. https://doi.org/10.1001/jama.280.5.443

65. Hollifield M, Warner TD, Lian N, Krakow B, Jenkins JH, Kesler J, Stevenson J, Westermeyer J. Measuring trauma and health status in refugees: A critical review. JAMA 2002;288, 611–621. https://doi.org/10.1001/jama.288.5.611.

For peer review only

For peer review only - http://bmjopen.bmj.com/site/about/guidelines.xhtml

Table 1. Overview of study measures

Assessment	*Screening Visit 1*	*Baseline, Enrollment Randomization: Visit 1*	*Intervention Session 1 Visit 2*	*Intervention Session 2 Visit 3*	*Intervention Session 3 Visit 4*	*Intervention Session 4 Visit 5*	*Intervention Session 5 Visit 6*	*Post-intervention Visit 7*	*3-month Follow-up Final Visit*
Informed Consent Form	X								
Screening tool	X								
Inclusion/Exclusion Criteria	X								
Demographics		X						X	X
Blood pressure		X						X	X
Bodyweight & height		X						X	X
Waist circumference		X						X	X
Hair samples		X							X
Stress, Anxiety & Depression		X						X	X
Family & Social Support		X						X	X
Coping Strategies		X						X	X
Self-efficacy		X						X	X
Family Conflict Resolution		X						X	X
Family Satisfaction		X							
Enrollment/Randomization		X							
Intervention Session and its assessment using fidelity form			X	X	X	X	X		
Adverse Events		X	X	X	X	X	X	X	X

For peer review only

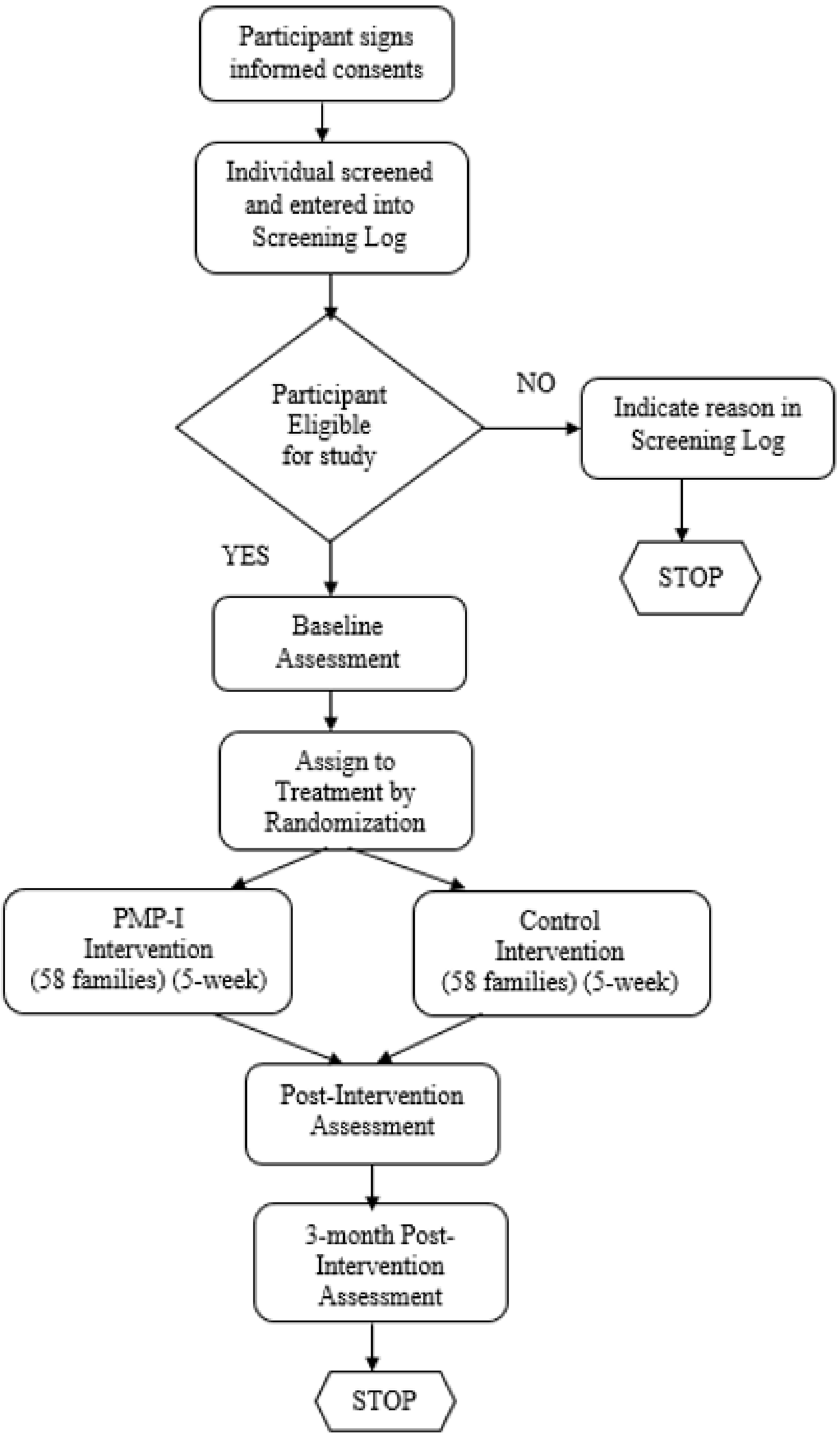

Figure 1 Flowchart of the study

Consent Form for Participation in a Research Study
University of Massachusetts Amherst

Researcher(s): Kalpana Poudel-Tandukar, Assistant Professor, College of Nursing
Study Title: Reducing stress, anxiety, and depressive symptoms via a family-centered preventative intervention for immigrants: A randomized controlled feasibility trial
Study Funder: National Institute of Mental Health, National Institutes of Health

---

1. WHAT IS THIS FORM?

"This form is called a Consent Form. It will give you information about the study so you can make an informed decision about participation in this research. We encourage you to take some time to think this over and ask questions now and at any other time. If you decide to participate, you will be asked to sign this form and you will be given a copy for your records."

2. WHAT ARE SOME OF THE IMPORTANT ASPECTS OF THIS RESEARCH STUDY THAT I SHOULD BE AWARE OF?

This study plans to implement mental health promotion program among Bhutanese adults resettled in Massachusetts through trained community interventionists in collaboration with church leaders. The overall objective of this study is to assess preliminary effect of a family-centered mental health promotion program (Problem Management Plus for Immigrants: PMP-I) to improve coping, family wellbeing, and social networking that would be helpful to reduce stress, anxiety, and depressive symptoms among Bhutanese adults resettled in Massachusetts. We will determine your eligibility by asking questions of Patient Health Questionnaire-9 (PHQ-9), a screening tool for depression. You are eligible to participate in this study as a primary study participant if your PHQ-9 score is 14 or below. If you are eligible and interested, we will take an informed consent prior to take about an hour interview before, after, and 3-month after program. We will request you to attend about 2-3 hours mental health promotion program each week for 5-week. You will be randomly assign into one of our program, PMP-I program or talk program using a community support service pamphlet. If you are assigned to talk program now, we will provide you PMP-I program after completion of 3-month follow up survey. You do not need to answer the questions, which you do not feel comfortable. Your information will be kept confidential and will not be documented with your name. Please find risks and benefits of study and other important details in the rest of the consent document.

3. WHY ARE WE DOING THIS RESEARCH STUDY?

Existing mental health interventions for immigrants are largely based on treatment models to improve the access and quality of care for those with diagnosed mental health problems, however culturally-tailored preventative behavioral interventions aimed at reducing stress and preventing mental health problems among immigrants are limited. Thus, we aim to develop, implement, and pilot test the preventive behavior intervention driven by community members to reduce stress and prevent mental health problems among immigrants.

4. WHO CAN PARTICIPATE IN THIS RESEARCH STUDY?

Bhutanese adult aged 18 years or older living in the Massachusetts with a score 14 or below on the Patient Health Questionnaire (PHQ-9) and are willing to participate in the study voluntarily will be requested to participate in the study as primary study participants. This includes parents and their adult children aged 18 and above. Bhutanese adults with PHQ-9 scores 15 or above or with clinically diagnosed mental disorders or taking psychiatric medications for any mental health problems will not be eligible to participate in this study as a primary study participant. However, all interested adult family members both

IRB OFFICE USE ONLY
University of Massachusetts Amherst-IRB
Protocol #: 1837
IRB Signature:

For peer review only

parents and their adult children, regardless of PHQ-9 score and mental health status, will be encouraged to participate in our mental health promotion intervention at family settings.

5. WHERE WILL THIS RESEARCH STUDY TAKE PLACE AND HOW MANY PEOPLE WILL PARTICIPATE?

This study will be conducted among Bhutanese adults resettled in the Massachusetts at their family settings. We plan to enroll 232 participants from 116 families in this study. The entire study is expected to complete in three year time period.

6. WHAT WILL I BE ASKED TO DO AND HOW MUCH TIME WILL IT TAKE?

If you agree to take part in this study, we will take your 5-minutes time asking short questions of Patient Health Questionnaire, which is a screening tool for depression. If you are eligible to participate in this study, we will take your informed consent first. Then, we will take about 45 to 60 minutes of your time for questionnaire survey before and after program and 3-month after program (thrice). The questionnaire consists of questions related to socio-demographic information, depression, perceived stress, family satisfaction, coping behavior, family/social support, family wellbeing, social network, sleep quality, and health information and program assessment information. We will also measure your body weight, height, waist circumference, and blood pressure. We will also take your 3-cm hair sample before and 3-month after program (twice). We request you not to write your name in the questionnaire. You may skip any question you feel uncomfortable answering. We will also take about two to three hours of your time to attend mental health promotion program each week for 5-week.

7. WILL BEING IN THIS RESEARCH STUDY HELP ME IN ANY WAY?

You may not directly benefit from this research; however, we hope that your participation in the study may find useful learning about mental health promotion activities.

8. WHAT ARE MY RISKS OF BEING IN THIS RESEARCH STUDY?

We believe there are minimal risks associated with this research study; however, a risk of breach of confidentiality always exists and we have taken the steps to minimize this risk as outlined in section 9 below. Some of the questions that we ask may put you in trouble or you may hesitate to answer, for example, questions on suicidal intention. You are free to skip such questions or also withdraw yourself from participating the whole study. As researchers, we are not qualified to provide counselling services and we will not be following up with you after this study. If you feel upset during the study, or find that some questions or aspects of the study triggered distress, you may contact the PI. The PI will assist you (in your native Nepali language) and help you to connect with mental health support services in coordination with the field supervisor. You may want to contact your counsellor at nearby health institutions such as Baystate Medical Center (Phone: 413-794-0000) and Caring Health Center (Phone: 413- 739-1100), Springfield. Field supervisors from community will provide necessary support to you for setting up your appointment with primary health care providers if needed.

9. HOW WILL MY PERSONAL INFORMATION BE PROTECTED?

Your privacy and confidentiality is important to us. The following procedures will be used to protect the confidentiality of your study records. All the information collected during the study will remain confidential. We will conduct survey in a private location, only allowing authorized research team members to meet with research participants. We will not record your name in the questionnaire. We will assign you numerical code that would be used in place of your name in all records to ensure confidentiality. We would like to assure you that your information will be kept confidential and will not be disclosed in your name in any of our record, report, publications, and presentations. Data will be stored securely in password protected computer and will be made available only to the PI. The PI will keep all study records, including any codes to your data, in a password protected computer. All signed consent documents will be

IRB OFFICE USE ONLY

University of Massachusetts Amherst-IRB
Protocol #: 1837
IRB Signature: Nancy C. Swett

For peer review only

stored securely and separately from the research data in the separate locked cabinet. All hardcopies of study materials will be stored securely in the locked cabinet of PI's office. Research records will be labeled with a code. A master key that links names and codes will be maintained in a separate and secure location. The master key and questionnaires will be completely destroyed six years after the close of the study. All electronic files containing identifiable information will be password protected. Any computer hosting such files will also have password protection to prevent access by unauthorized users. Only the PI will have access to the passwords. At the conclusion of this study, we plan to publish the study findings. Information will be presented in summary format and you will not be identified in any publications or presentations.

The hair sample will be stored in a clean dry white envelope with your ID number on top of the envelope. The hair samples will be stored in the College of Nursing's laboratory during the data collection process. At the end of survey, the collected hair samples will be sent to the laboratory in UMass Amherst. Hair samples will be processed in the laboratory for cortisol measurement. If sample remains after measurement procedure completion, it will be disposed according to the safety rules and regulation of the laboratory.

For this study you will be assigned a global unique identifier (GUID). This GUID is generated as a subject ID that allows researchers to share raw data such as number or percentage specific to a study participant without exposing personally identifiable information. The GUID is made up of random alpha-numeric characters and is NOT generated from personally identifiable information or protected health information. This identifier will be kept separate from your paper consent file and will be stored in a password protected electronic file. Descriptive/raw data will be submitted semi-annually. Access to raw data used in the proposed project will be considered for sharing in compliance with the NIH Grants Policy on Sharing Unique Research Resources. Any raw data to be released for sharing will not contain identifiers (such as name, address, birthdate and phone number) of the study participants.

During and after the study, we will send deidentified study data to the National Institute of Mental Health Data Archive (NDA). Experts at the NIH who know how to keep your data safe. The study researchers will make every attempt to protect your identity. The study data provided to NDA may help researchers around the world learn more about mental health and how to help others who have problems with mental health. NIMH will also report to Congress and on its website about the different studies using NDA data. You will not be contacted directly about the study data you contributed to NDA. You may decide now or later that you do not want your study data to be added to the NDA. You can still participate in this research study even if you decide that you do not want your data to be added to the NDA.

This research is covered by a Certificate of Confidentiality from the National Institutes of Health. The researchers with this Certificate may not disclose or use information, documents, or hair samples that may identify you in any federal, state, or local civil, criminal, administrative, legislative, or other actions.

A description of this study will be available on http://www.ClinicalTrials.gov. This Web site will not include information that can identify you. At most, the Website will include a summary of the results, when they are available. You can search this Web site at any time. The registration number for this study is NCT04453709.

10. WHAT IF THERE IS AN UNEXPECTED FINDING ON TESTS CONDUCTED ON MY HAIR SAMPLES?

The investigators for this research project are not licensed or trained diagnosticians or clinicians. The testing performed in this project is not intended to find abnormalities, and the data collected do not comprise a diagnostic or clinical study thus we would not be returning/sharing the results of tests that are conducted on your hair samples.

11. WILL MY INFORMATION (HAIR SAMPLES OR PRIVATE INFORMATION) BE USED FOR RESEARCH IN THE FUTURE?

Your information or hair samples will not be used or distributed for future research studies even if identifiers are removed.

IRB OFFICE USE ONLY

University of Massachusetts Amherst-IRB
Protocol #: 1837
IRB Signature: Nancy C. Swett

For peer review only

12. WILL I BE GIVEN ANY MONEY OR OTHER COMPENSATION FOR BEING IN THIS RESEARCH STUDY?

You will be interviewed about 45 to 60 minutes for three times (before and after program and 3-month after program). We will provide total $25 cash for each participant after completion of each survey.
After baseline survey: $25 per individual
After post-intervention survey: $25 per individual
After 3-month follow up survey: $25 per individual
After intervention: $50 per family ($10 per session)
Exercise mat: One per individual

13. WHO CAN I TALK TO IF I HAVE QUESTIONS?

Take as long as you like before you make a decision. We will be happy to answer any question you have about this study. If you have further questions about this project or if you have a research-related problem, you may contact the researcher, [Dr. Kalpana Poudel Tandukar; email: kalpana@umass.edu; Tel: 1-413-545-5095). If you have any questions concerning your rights as a research subject, you may contact the University of Massachusetts Amherst Human Research Protection Office (HRPO) at 1-413-545-3428 or humansubjects@ora.umass.edu.

14. WHAT HAPPENS IF I SAY YES, BUT I CHANGE MY MIND LATER?

You do not have to be in this study if you do not want to. If you agree to be in the study, but later change your mind, you may drop out at any time. There are no penalties or consequences of any kind if you decide that you do not want to participate. You will be notified of all significant new findings during the course of the study that may affect your willingness to continue.

15. WHAT IF I AM INJURED?

The University of Massachusetts does not have a program for compensating subjects for injury or complications related to human subjects research, but the study personnel will assist you in getting treatment.

16. SUBJECT STATEMENT OF VOLUNTARY CONSENT

When signing this form I am agreeing to voluntarily enter this study. I have had a chance to read this consent form, and it was explained to me in a language which I use. I have had the opportunity to ask questions and have received satisfactory answers. I have been informed that I can withdraw at any time. A copy of this signed Informed Consent Form has been given to me.

______________________	______________________	__________
Participant Signature:	Print Name:	Date:

By signing below I indicate that the participant has read and, to the best of my knowledge, understands the details contained in this document and has been given a copy.

______________________	______________________	__________
Signature of Person Obtaining Consent	Print Name:	Date:

IRB OFFICE USE ONLY

University of Massachusetts Amherst-IRB
Protocol #: 1837
IRB Signature: Nancy C. Swett

For peer review only

SPIRIT

STANDARD PROTOCOL ITEMS: RECOMMENDATIONS FOR INTERVENTIONAL TRIALS

SPIRIT 2013 Checklist: Recommended items to address in a clinical trial protocol and related documents*

Section/item	Item No	Description	Addressed on page number
**Administrative information**			
Title	1	Descriptive title identifying the study design, population, interventions, and, if applicable, trial acronym	_____1_________
Trial registration	2a	Trial identifier and registry name. If not yet registered, name of intended registry	_____3________
	2b	All items from the World Health Organization Trial Registration Data Set	_____NA_______
Protocol version	3	Date and version identifier	_____NA______
Funding	4	Sources and types of financial, material, and other support	____24-25______
Roles and responsibilities	5a	Names, affiliations, and roles of protocol contributors	____25________
	5b	Name and contact information for the trial sponsor	____24-25______
	5c	Role of study sponsor and funders, if any, in study design; collection, management, analysis, and interpretation of data; writing of the report; and the decision to submit the report for publication, including whether they will have ultimate authority over any of these activities	____24-25______
	5d	Composition, roles, and responsibilities of the coordinating centre, steering committee, endpoint adjudication committee, data management team, and other individuals or groups overseeing the trial, if applicable (see Item 21a for data monitoring committee)	___ 19__________

For peer review only

**Introduction**			
Background and rationale	6a	Description of research question and justification for undertaking the trial, including summary of relevant studies (published and unpublished) examining benefits and harms for each intervention	___5 - 7_______
	6b	Explanation for choice of comparators	____13_________
Objectives	7	Specific objectives or hypotheses	____6 - 7_______
Trial design	8	Description of trial design including type of trial (eg, parallel group, crossover, factorial, single group), allocation ratio, and framework (eg, superiority, equivalence, noninferiority, exploratory)	____7 - 8______
**Methods: Participants, interventions, and outcomes**			
Study setting	9	Description of study settings (eg, community clinic, academic hospital) and list of countries where data will be collected. Reference to where list of study sites can be obtained	____ 7_________
Eligibility criteria	10	Inclusion and exclusion criteria for participants. If applicable, eligibility criteria for study centres and individuals who will perform the interventions (eg, surgeons, psychotherapists)	____8 - 9_______
Interventions	11a	Interventions for each group with sufficient detail to allow replication, including how and when they will be administered	____11 - 13_____
	11b	Criteria for discontinuing or modifying allocated interventions for a given trial participant (eg, drug dose change in response to harms, participant request, or improving/worsening disease)	___ 19 - 21______
	11c	Strategies to improve adherence to intervention protocols, and any procedures for monitoring adherence (eg, drug tablet return, laboratory tests)	___ 19 - 21______
	11d	Relevant concomitant care and interventions that are permitted or prohibited during the trial	____NA________
Outcomes	12	Primary, secondary, and other outcomes, including the specific measurement variable (eg, systolic blood pressure), analysis metric (eg, change from baseline, final value, time to event), method of aggregation (eg, median, proportion), and time point for each outcome. Explanation of the clinical relevance of chosen efficacy and harm outcomes is strongly recommended	___13 - 16______
Participant timeline	13	Time schedule of enrolment, interventions (including any run-ins and washouts), assessments, and visits for participants. A schematic diagram is highly recommended (see Figure)	___ 34_________

For peer review only - http://bmjopen.bmj.com/site/about/guidelines.xhtml

Sample size	14	Estimated number of participants needed to achieve study objectives and how it was determined, including clinical and statistical assumptions supporting any sample size calculations	___9 - 10_______
Recruitment	15	Strategies for achieving adequate participant enrolment to reach target sample size	___9 - 10_______

Methods: Assignment of interventions (for controlled trials)

Allocation:

Sequence generation	16a	Method of generating the allocation sequence (eg, computer-generated random numbers), and list of any factors for stratification. To reduce predictability of a random sequence, details of any planned restriction (eg, blocking) should be provided in a separate document that is unavailable to those who enrol participants or assign interventions	_____10________
Allocation concealment mechanism	16b	Mechanism of implementing the allocation sequence (eg, central telephone; sequentially numbered, opaque, sealed envelopes), describing any steps to conceal the sequence until interventions are assigned	_____10_______
Implementation	16c	Who will generate the allocation sequence, who will enrol participants, and who will assign participants to interventions	_____10________
Blinding (masking)	17a	Who will be blinded after assignment to interventions (eg, trial participants, care providers, outcome assessors, data analysts), and how	_____10________
	17b	If blinded, circumstances under which unblinding is permissible, and procedure for revealing a participant's allocated intervention during the trial	____NA_______

Methods: Data collection, management, and analysis

Data collection methods	18a	Plans for assessment and collection of outcome, baseline, and other trial data, including any related processes to promote data quality (eg, duplicate measurements, training of assessors) and a description of study instruments (eg, questionnaires, laboratory tests) along with their reliability and validity, if known. Reference to where data collection forms can be found, if not in the protocol	___16 - 19______
	18b	Plans to promote participant retention and complete follow-up, including list of any outcome data to be collected for participants who discontinue or deviate from intervention protocols	___10, 16 - 17___

Data management	19	Plans for data entry, coding, security, and storage, including any related processes to promote data quality (eg, double data entry; range checks for data values). Reference to where details of data management procedures can be found, if not in the protocol	__17 - 18_______
Statistical methods	20a	Statistical methods for analysing primary and secondary outcomes. Reference to where other details of the statistical analysis plan can be found, if not in the protocol	__18 - 19_______
	20b	Methods for any additional analyses (eg, subgroup and adjusted analyses)	__18 - 19_______
	20c	Definition of analysis population relating to protocol non-adherence (eg, as randomised analysis), and any statistical methods to handle missing data (eg, multiple imputation)	__18 - 19_______
**Methods: Monitoring**			
Data monitoring	21a	Composition of data monitoring committee (DMC); summary of its role and reporting structure; statement of whether it is independent from the sponsor and competing interests; and reference to where further details about its charter can be found, if not in the protocol. Alternatively, an explanation of why a DMC is not needed	___19__________
	21b	Description of any interim analyses and stopping guidelines, including who will have access to these interim results and make the final decision to terminate the trial	___19__________
Harms	22	Plans for collecting, assessing, reporting, and managing solicited and spontaneously reported adverse events and other unintended effects of trial interventions or trial conduct	__ 20 - 21_______
Auditing	23	Frequency and procedures for auditing trial conduct, if any, and whether the process will be independent from investigators and the sponsor	___NA________
**Ethics and dissemination**			
Research ethics approval	24	Plans for seeking research ethics committee/institutional review board (REC/IRB) approval	__24_________
Protocol amendments	25	Plans for communicating important protocol modifications (eg, changes to eligibility criteria, outcomes, analyses) to relevant parties (eg, investigators, REC/IRBs, trial participants, trial registries, journals, regulators)	__19___________

Consent or assent	26a	Who will obtain informed consent or assent from potential trial participants or authorised surrogates, and how (see Item 32)	____9___________
	26b	Additional consent provisions for collection and use of participant data and biological specimens in ancillary studies, if applicable	____NA__________
Confidentiality	27	How personal information about potential and enrolled participants will be collected, shared, and maintained in order to protect confidentiality before, during, and after the trial	____17 - 18______
Declaration of interests	28	Financial and other competing interests for principal investigators for the overall trial and each study site	__ 24__________
Access to data	29	Statement of who will have access to the final trial dataset, and disclosure of contractual agreements that limit such access for investigators	____24___________
Ancillary and post-trial care	30	Provisions, if any, for ancillary and post-trial care, and for compensation to those who suffer harm from trial participation	____NA__________
Dissemination policy	31a	Plans for investigators and sponsor to communicate trial results to participants, healthcare professionals, the public, and other relevant groups (eg, via publication, reporting in results databases, or other data sharing arrangements), including any publication restrictions	__17 - 18_______
	31b	Authorship eligibility guidelines and any intended use of professional writers	____NA__________
	31c	Plans, if any, for granting public access to the full protocol, participant-level dataset, and statistical code	_____**24**_________
**Appendices**			
Informed consent materials	32	Model consent form and other related documentation given to participants and authorised surrogates	______________
Biological specimens	33	Plans for collection, laboratory evaluation, and storage of biological specimens for genetic or molecular analysis in the current trial and for future use in ancillary studies, if applicable	______________

*It is strongly recommended that this checklist be read in conjunction with the SPIRIT 2013 Explanation & Elaboration for important clarification on the items. Amendments to the protocol should be tracked and dated. The SPIRIT checklist is copyrighted by the SPIRIT Group under the Creative Commons "Attribution-NonCommercial-NoDerivs 3.0 Unported" license.

For peer review only - http://bmjopen.bmj.com/site/about/guidelines.xhtml
