# Supplementary material for: Peer-led family-centred problem management plus for immigrants (PMP-I) for mental health promotion among immigrants in USA: protocol for a pilot, randomised controlled feasibility trial
Source: BMJ Open. 2022 Apr 28;12(5):e061353. doi: 10.1136/bmjopen-2022-061353 (PMC9097550; doi:10.1136/bmjopen-2022-061353)
Supplement: Reviewer comments [file bmjopen-2022-061353.reviewer_comments.pdf]

PEER REVIEW HISTORY

BMJ Open publishes all reviews undertaken for accepted manuscripts. Reviewers are asked to complete a checklist review form (**http://bmjopen.bmj.com/site/about/resources/checklist.pdf**) and are provided with free text boxes to elaborate on their assessment. These free text comments are reproduced below.

ARTICLE DETAILS

**TITLE (PROVISIONAL)**	Peer-led family-centered Problem Management Plus for Immigrants (PMP-I) for mental health promotion among immigrants in the United States: protocol for a pilot, randomized controlled feasibility trial
**AUTHORS**	Poudel-Tandukar, Kalpana; Jacelon, Cynthia; Martell, Christopher; Poudel, Krishna; Rai, Shan; Ramdam, Razu; Laws, Holly; Meyer, Jerrold; Bertone-Johnson, Elizabeth; Hollon, Steven

This article was not externally reviewed at BMJ Open. Protocols that have been independently assessed prior to submission to BMJ Open are usually fast-tracked to publication on the grounds that further substantial changes will not be possible. This independent assessment will usually be external, independent review for both a substantial grant award from a non-commercial or government funder and ethics approval.
